# Precision‐Engineered Silver Single‐Atom Carbon Dot Nanozymes for Theranostic Management of Acute Kidney Injury

**DOI:** 10.1002/advs.202519393

**Published:** 2026-01-15

**Authors:** Tianle Tang, Jun Zhang, Yan Wang, Guoping Chen, Kehui Yuan, Yuan He, Chenchen Li, Sumaira Hanif, Yang Yang, Yanli Wang, Pir Muhammad

**Affiliations:** ^1^ NHC Key Laboratory of Tropical Disease Control, School of Life Sciences and Medical Technology Hainan Medical University Haikou Hainan China; ^2^ International Joint Research Center of Human‐machine Intelligent Collaborative for Tumor Precision Diagnosis and Treatment of Hainan Province Engineering Research Center of Tropical Medicine Innovation and Transformation of Ministry of Education School of Pharmacy Hainan Academy of Medical Sciences Hainan Medical University Haikou Hainan China; ^3^ The First Affiliated Hospital of Hainan Medical University Hainan Medical University Haikou China

**Keywords:** acute kidney injury, antioxidant nanozyme, ROS scavenging, single‐atom Ag, theranostic

## Abstract

Overproduction of reactive oxygen species (ROS) is a key pathogenic feature in acute kidney injury (AKI), leading to rapid decline in renal function with high mortality rates that call for effective antioxidant therapies. Herein, we present triphenylphosphonium‐functionalized carbon dots supported by single‐atom silver (T‐Ag_SA_‐CDs) that integrate fluorescent antioxidant nanozymes for the accurate ROS scavenging and real‐time bioimaging of AKI. By anchoring silver in single‐atom and sub‐nanocluster states on a carbon dot matrix, T‐Ag_SA_‐CDs exhibit exceptional superoxide dismutase (SOD)‐ and glutathione peroxidase (GPx)‐like activities, surpassing traditional nanozymes in ROS neutralization efficiency. Density functional theory (DFT) calculations disclose a low‐energy reaction pathway common to both nitrogen‐doped carbon dots (N‐CDs) and Ag_SA_‐CDs, clarifying the mechanism behind their dual SOD‐ and GPx‐mimetic activities. The biocompatible N‐CDs platform guarantees the stability, minimizes Ag^+^‐associated toxicity, and enhances catalytic performance through synergistic Ag_SA_‐CD interactions. Targeting mitochondria through triphenylphosphonium functionalization facilitates site‐specific antioxidant protection, demonstrating robust therapeutic efficacy in a cisplatin‐induced AKI mouse model. Additionally, the intrinsic fluorescence of T‐Ag_SA_‐CDs facilitates non‐invasive monitoring of biodistribution and renal accumulation, promotes the recovery of damaged kidney tissue, alleviates oxidative stress and post‐cure assessment, and offers a self‐reported theranostic platform, while also aiming to improve its clinical application.

## Graphical abstract

Mitochondria‐targeted silver single‐atom carbon dot (T‐AgSA‐CDs) nanozymes enable dual SOD/GPx‐like ROS scavenging, real‐time imaging, and therapeutic effects for acute kidney injury (AKI). Atomically dispersed Ag sites synergize with carbon dots to restore renal redox hemostasis, protect mitochondrial function, mitigate inflammation, facilitate post‐recovery assessment in cisplatin‐induced AKI, thereby establishing a self‐reported theranostic nanozyme platform through modulation of the NF‐κB, KEAP1, and GPX4/HO‐1 pathways.

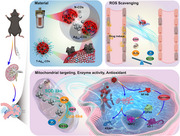

## 1 Introduction

Overproduction of reactive oxygen species (ROS) is an important pathological feature of many diseases, including acute kidney injury (AKI), a critical clinical syndrome characterized by the sudden loss of renal function and high mortality [1, 2]. ROS are required for normal cellular signaling; however, excessive accumulation of ROS may lead to oxidative stress, which will result in damage to DNA, protein denaturation, and lipid peroxidation that eventually induce apoptosis and interruption of visceral function [3, 4, 5]. AKI is marked by a fast deterioration in renal function, frequently induced by an increase in intrarenal ROS levels after toxic assaults or ischemic occurrences [6, 7]. Despite the high morbidity, prevalence, and death rates associated with AKI, efficient therapeutic strategies that intervene directly and effectively to diminish oxidative stress remain a significant clinical challenge, emphasizing the need for novel included antioxidant agents [8, 9].

Nanozymes, a kind of nanomaterials possessing enzyme‐like activities and mimetic functions, inspired the development of ROS scavenging therapeutics through introducing novel solutions to diseases associated with oxidative stress [10, 11, 12]. Compared to their natural counterpart, nanozymes are more stable, cheaper, and possess adjustable catalytic activities [13, 14, 15]. Of these, nanozymes that mimic the vital antioxidant enzymes such as superoxide dismutase (SOD), catalase (CAT), and glutathione peroxidase (GPx) have demonstrated outstanding potential in scavenging of superoxide anions (O_2_•^−^) and hydrogen peroxide (H_2_O_2_), respectively [16, 17, 18]. The SOD, CAT, and GPX are the most important components of the intricate antioxidant defense mechanism of the body, which have critical functions in the normal metabolism of the body, mainly through mitochondrial energy generation pathways [19, 20]. Nevertheless, the search for highly efficient, multifunctional, and biosafe antioxidant nanozymes that exhibit strong multienzyme activities under physiological conditions is still challenging to date. A critical strategy to enhance performance is engineering the nanozyme at the sub‐nanometer scale, where the maximized exposure of active sites and unique electronic structures can lead to unprecedented catalytic activity [21, 22, 23].

Silver (Ag) has been explored for its remarkable antimicrobial and catalytic properties. [24, 25, 26] However, the application of Ag in antioxidant nanozymes has been reported less frequently, as it is prone to problems such as toxicity that is induced by Ag^+^ ions and instability of the particle [27, 28]. These issues can be overcome by fine‐tuning the Ag through ultrasmall nanoclusters or atomic‐level engineering, which is conducive to stabilizing these clusters over a biocompatible support. Carbon dots (CDs) have emerged as an ideal platform for such a purpose. CDs exhibited superior water dispersibility, minimal toxicity, and abundant surface functional groups that serve as anchors for metal clusters, thereby inhibiting aggregation and improving catalytic performance through synergistic interactions [29, 30]. Furthermore, the inherent fluorescence properties of CDs provide a unique opportunity for bioimaging and real‐time monitoring of treatment efficacy, a feature highly desired for theranostic applications [31]. Recently, CDs were extensively used as an antioxidant nanozyme for defense management. Fan et al. developed red‐emissive CDs with excellent SOD‐like activity (>10 000 U mg^−1^) [32]. However, their low quantum yield (QY) with undoped capability restricted them for bioimaging uses in in vivo setups. Liu et al. developed C‐dot‐based SOD‐nanozyme (>4000 U mg^−1^) that exhibits red fluorescence with an emission wavelength of 683 nm, with in vivo bioimaging capability [33]. Though the enzyme mimic with a superb bioimaging response makes it superior, the SOD activity does not match the requirement to give an antioxidant nanozymatic response, with these CDs‐based nanozymes having stable and long‐term imaging capacity, which opens up a new opportunity to develop a novel multienzyme antioxidant mimic.

**SCHEME 1:**
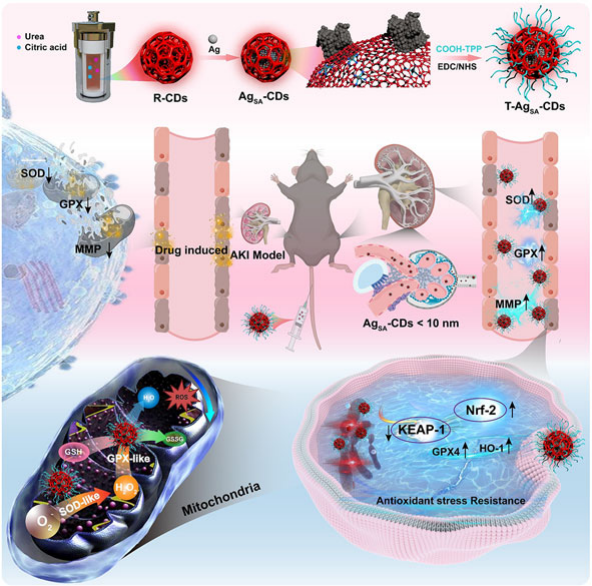
Schematic illustration of the synthesis and therapeutic mechanism of mitochondria‐targeted T‐Ag_SA_‐CDs for AKI therapy. In cisplatin‐induced AKI, T‐Ag_SA_‐CDs (<10 nm) scavenge ROS via SOD‐ and GP_X_‐like activities, restore mitochondrial membrane potential (Ψm), and activate the KEAP‐1/Nrf2 pathway, thereby upregulating HO‐1 and GPX4 to enhance antioxidant defense and alleviate renal injury.

In this study, we present a rational design and synthesis of a novel antioxidant nanozyme: the carbon dot–supported silver single‐atom nanozyme (Ag_SA_‐CDs). We establish an extremely stable and multifunctional catalytic platform that exhibits superior SOD‐ and GPX‐like activities for efficiently scavenging ROS by trapping Ag in single‐atom and sub‐nanocluster states on a CDs matrix (Scheme 1). This strategy reduces the toxicity that larger Ag nanoparticles usually have while taking advantage of the synergistic effects of Ag_SA_ and CDs to make the catalyst work better. The resulting nanozyme shows strong therapeutic effects in a cisplatin‐induced AKI mouse model and potent multi‐enzyme mimetic activity and ROS clearance in vitro. Significantly, the functionalization of triphenylphosphonium (T) to Ag_SA_‐CDs (T‐Ag_SA_‐CDs) improves this system for targeting the mitochondria, which allows site‐specific antioxidation protection and offers a comprehensive perspective on systemic targeted therapies. In addition to therapeutic applications, the intrinsic fluorescence of the T‐Ag_SA_‐CDs nanozyme offers a self‐reporting capability, which allows for real‐time monitoring of biodistribution, renal accumulation, clearance, and assessment of recovery post‐treatment. In this work, Ag_SA_‐CDs are the first theranostic nanozyme that integrates precise ROS modulation through multienzyme mimicry and non‐invasive bioimaging. Due to these merits, this nanoplatform opens up new possibilities for next‐generation antioxidant nanozymes disease theranostic capability that connects nanomedicine and clinical translation.

## 2 Results and Discussion

### 2.1 Design and Characterization of Ag_SA_‐CDs/N‐CDs Nanozymes

**FIGURE 1:**
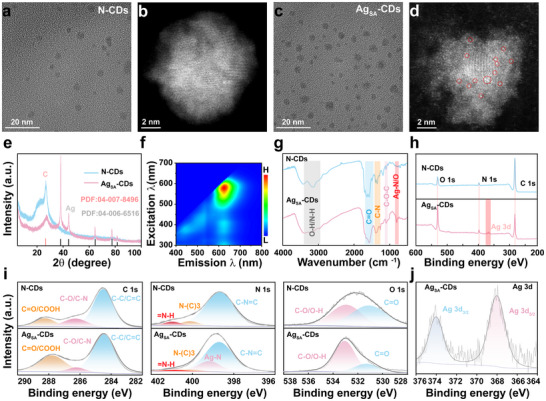
Characterization of CDs with and without supported Ag_SA_ nanozymes. TEM of N‐CDs (a) and Ag_SA_‐CDs (c) images. Ac‐HAADF‐STEM image of N‐CDs (b) and Ag_SA_‐CDs (d) with red circle showing the single atom Ag particles. e) XRD spectra (with respective PDF JCPDS No.) of the N‐CDs and Ag_SA_‐CDs nanozyme. f) Excitation‐emission 3D matrices of the Ag_SA_‐CDs nanozyme. g) FTIR spectra of N‐CDs and Ag_SA_‐CDs. h) XPS characterizations of N‐CDs and Ag_SA_‐CDs with full survey XPS spectra. i) C1s scan, N1s scan, O1s scan of N‐CDs and Ag_SA_‐CDs; (j) high resolution convoluted Ag3d XPS spectra of Ag_SA_‐CDs.

Nitrogen‐doped CDs (N‐CDs) were synthesized using a single‐step hydrothermal method in which citric acid and urea were used as a source of carbon (C) and nitrogen (N), respectively. The incorporation of silver (Ag) as a dopant was achieved using a modified two‐step method, as reported in previous studies [34], which enhanced the physicochemical properties of the N‐CDs. The doping strategy is likely to enhance the fluorescence and catalytic performance of the carbon dots, making them suitable for widespread advanced applications in biomedicine and photocatalysis [35]. Figure 1a,c represent transmission electron microscopy (TEM) images of N‐CDs and Ag_SA_‐CDs with and without Ag loadings. The N‐CDs and Ag_SA_‐CDs exhibited uniform spherical size distribution and were well dispersed, with an average diameter of 7.07 ± 1.63 and 8.49 ± 1.71 nm, respectively. The TEM image of N‐CDs lacks a prominent, well‐defined lattice structure (Figure 1b). Furthermore, the TEM image (Figure 1d) indicated that the lattice spacing is ≈0.24 nm, which corresponds to the (111) crystal face of Ag (JCPDS No. 04–0783). TEM results indicate that the substrate has multiple forms of Ag; however, atomic resolution reveals these as either isolated or clustered groups of Ag atoms. Therefore, spherical aberration‐corrected scanning transmission electron microscopy (Ac‐STEM) was employed to analyze the Ag_SA_‐CDs with greater precision. As shown in Figure 1d and Figure S1, the aberration‐corrected high‐angle toroidal dark field image‐scanning transmission electron microscopy (Ac‐HAADF‐STEM) images demonstrated that Ag is present not only as a nanocluster of Ag but also in an atomically dispersed state. The results presented indicated that the Ag_SA_‐CDs sample is composed of individual Ag atoms and clusters measuring a few nm in size (3–5 nm), modified with an N‐doped amorphous carbon framework of CDs. Thus, to elucidate the precise role of doping with metals in the enzyme‐mimicking activity that enhances the antioxidant effect, metal‐free N‐CDs nanozymes were synthesized utilizing the same methodology. The Ag loading did not influence the size, geometry, or photoluminescence (PL) properties, indicating that the N‐CDs exhibited excellent stability. These samples were measured for Ag content quantitatively by inductively coupled plasma optical emission spectrometer (ICP‐OES) (Table S1). The Ag_SA_‐CDs nanozyme had a total loading of Ag of 5.8 wt%, demonstrating the integration of Ag into the N‐CDs structure, which is beneficial to enhance both the catalytic efficiency and stability of antioxidant nanozyme. The high‐metal‐density loading on the support offers significant potential for atomic utilization and ROS scavenging in Ag_SA_‐CDs.

The X‐Ray Diffraction (XRD) patterns of N‐CDs and Ag_SA_‐CDs show a broad diffraction peak centered at about 24°, which is typical of amorphous carbon (002) planes (Figure 1e), confirming that the N‐CDs framework is made of graphite carbon. Upon Ag single‐atom incorporation, additional weak diffraction peaks corresponding to metallic silver (PDF# 04‐007‐8496 and 04‐006‐6516) appear, indicating successful anchoring of Ag sites. The lack of sharp crystalline peaks beyond these demonstrates that silver is present in highly dispersed or sub‐nanometer states, rather than in the form of large nanoparticles.

The optical properties of Ag_SA_‐CDs and N‐CDs were characterized using UV–vis spectroscopy and fluorescence spectroscopy. As illustrated in Figure S2, both Ag_SA_‐CDs and N‐CDs exhibit a prominent absorption band around 560 nm in their UV–vis spectra. In the Ag_SA_‐CDs, one typical absorption peak of Ag at 410 nm corresponds to the surface plasmon resonance (SPR) of Ag or single‐atom Ag sites interacting with the sp^2^ aromatic graphitic framework bond. In single‐atom Ag, this peak may result from localized electronic transitions or charge transfer between Ag atoms and the carbon dot matrix. The 560 nm peak, on the other hand, is related to the n–π^*^ transitions or lower‐energy states of the carbon dots. In N‐CDs, only one peak was observed. Similarly, the full‐scale fluorescence (FL) intensity of N‐CDs and Ag_SA_‐CDs demonstrates that Ag doping enhances the FL intensity due to improved electronic properties and surface stabilization (Figure S3). However, the stability of the signal intensity in Ag_SA_‐CDs shows slight variations compared to N‐CDs, likely due to the influence of Ag atoms on the photophysical properties. The fluorescence spectroscopy of Ag_SA_‐CDs/N‐CDs exhibits excitation‐dependent emission that ranges from 400 to 580 nm, with a consistent emission peak observed at 640 nm (Figure 1f; Figure S4). The emission wavelength of Ag_SA_‐CDs/N‐CDs exhibited a unique luminescent center in both materials, indicating that the Ag doping did not result in significant variations in PL intensity. Due to protonation‐deprotonation reactions changing their surface functional groups, pH affects N‐CD and Ag_SA_‐CD fluorescence intensity (Figure S5). As it was considered that the intrinsic fluorescence of CDs is very sensitive to pH because the surface functional groups may undergo protonation and deprotonation [36], we investigated the pH effect on the intrinsic fluorescence. As the pH drops from 7.4 to 4.5, N‐CDs show a noticeable reduction in emission and a red shift of about 10–20 nm (Figure S6a). On the other hand, coordination of a single atom of Ag to nitrogen sites in Ag_SA_‐CDs effectively shields these groups, which dramatically increases their pH tolerance. The emission intensity and λmax stay almost the same across the same pH range (Figure S6b). Adding a T‐Ag_SA_‐CDs variant further locks the surface charge, making fluorescence nearly independent of pH, even in DMEM culture medium (Figure S6c). Hence, this optical stability under physiological‐to‐acidic conditions makes T‐Ag_SA_‐CDs better choices for reliable bioimaging and sensing applications.

The chemical composition present in Ag_SA_‐CDs and N‐CDs nanzoymes were analyzed by spectroscopic methods such as Fourier‐transform infrared (FTIR) and X‐ray photoelectron spectroscopy (XPS). The FTIR spectra of the Ag_SA_‐CDs/N‐CDs (Figure 1g) showed different types of O and N containing multiple functional groups on their surface, including carboxyl (υ(C═O)), and C─C (sp^2^) peaks at 1670–1750 cm^−1^, carbon‐nitrogen (C‐N) at 1500–1630 cm^−1^, and hydroxyl (υ(O–H), υ(N‐H) at 3200–3450 cm^−1^). The Ag‐N/O peak is solely observed in Ag_SA_‐CDs within the range of 530 cm^−1^, while missing on N‐CDs. We examined the intricate surface functionalities available on Ag‐N/O‐CDs through XPS analysis. The XPS spectra of N‐CDs and Ag_SA_‐CDs in Figure 1h and Table S2 confirmed the elemental composition by C1s, O1s, N1s, and Ag3d peaks at approximately 284.65, 531.31, 398.98, and 368.3–374 eV, respectively. The above results indicate that the N and Ag were successfully doped into the CDs frameworks. The higher percentages of composition of O1s and N1s in Ag_SA_‐CDs (23.3 and 11.26, at.%) relative to N‐CDs (19.86 and 9.99 wt.%) further support the ameliorated surface functionalization. In contrast, the identification of Ag3d (5.37 at.%) verifies the presence of silver doping in Ag_SA_‐CDs. The high‐resolution XPS spectra (deconvoluted) also showed many Ag‐binding sites coordinated by C and N atoms. In the high‐resolution C1s XPS spectrum (Figure 1i), three obvious peaks at 288.28, 286.6, and 284.8 eV are attributed to the signals of C═O/COOH, C─N/C─O, and C─C/C═C groups, respectively. These peaks indicate the different C‐binding sites. Nevertheless, there is a small enhancement and shift of the C═O/COOH peak from Ag_SA_‐CDs after Ag‐doped (from around 288.28 eV down to ∼287 eV) in the C1s spectrum. This change is an indication of changes in the electronic environment of C due to interaction with Ag single atoms that will modify surface chemistry and reactivity.

The N1s XPS spectrum of Ag_SA_‐CDs exhibited various peaks at 398.67, 399.18, 400.10, 400.98, and 402.5 eV with associated with graphitic N, pyrrolic N, pyridinic N/Ag–N, and oxidized, respectively (Figure 1i). In contrast, the N1s spectrum of N‐CDs shows peaks at 398.67, 400.10, and 400.98 eV, which are assigned as pyridinic N, tertiary N, and pyrrolic‐N (NH_2_/pyrrolic) groups, respectively. The Ag‐N peak at 399.18 eV is only present in the Ag_SA_‐CDs and further confirms that the single‐atom Ag was coordinated with N. The marginal changes of these peaks between N‐CDs and Ag_SA_‐CDs, which imply that the introduced Ag atoms facilitate the modulation of conformation of N‐bonding, promoting better charge transfer as well as stability. Similarly, the O1s spectra for N‐CDs and Ag_SA_‐CDs have peaks at ∼531.14 eV (C = O) followed by a C‐O/O‐H region, but shifted from 532.93 eV in N‐CDs to 533.07 eV in Ag_SA_‐CDs (Figure 1i). This displacement of the C‐O/O‐H peak indicates modified O‐containing functional groups, likely in response to Ag‐induced alterations of coordination or surface polarity. The unaltered C═O peak reflects the stability of the carbonyl groups upon Ag doping. The Ag3d XPS spectrum of the Ag_SA_‐CDs exhibits typical peaks located at 374.02 eV (Ag^0^ 3d_5/2_) and 368.02 eV (Ag^0^ 3d_3/2_), which can be assigned to metallic Ag^0^ single atoms in the C around N framework (Figure 1j). Lack of oxidized Ag species indicates that Ag was retained in metallic states, which is necessary for the case of high catalytic performances required applications as well as good electrical conductivity. The XPS data unambiguously indicate that doping of Ag induces drastic chemical and electronic modifications into the N‐CDs structure, primarily attributed to Ag‐N coordination as well as subtle changes in C and O environments. These structural feature supports the assumption that atomic dispersion of Ag promotes the catalytic and antioxidant activity of nanozymes.

Surface functionalization of the targeting T‐moiety to Ag_SA_‐CDs was carried out via carbodiimide crosslinker reaction, as described elsewhere with minor modifications [34]. Characteristic signals of N‐CDs and Ag_SA_‐CDs were observed from their 1H NMR spectra, consistent with the highly carbonized and amorphous nature (Figure S7). The spectrum of T‐Ag_SA_‐CDs, on the other hand, shows distinctive proton resonances that match well with the characteristic peaks of the T ligand, indicating successful surface modification of Ag_SA_‐CD. Consequently, we analyzed the morphology of the conjugated T‐Ag_SA_‐CDs using TEM, revealing a uniform nanoparticle structure with no signs of agglomeration, indicating improved stability (Figure S8). We then compared the stability of N‐CDs, Ag_SA_‐CDs, and T‐Ag_SA_‐CD nanozymes by systematically investigating them using dynamic light scattering (DLS) analysis for particle size distribution and polydispersity index (PDI), as shown in Figure S9a,b. DLS and PDI data clearly showed that after various ROS conditions and pH effect, our nanoparticles were stable with no loss of metal leaching at pH 4.5, 6.5, and 7.4, and cell culturing medium DMEM and Fetal bovine serum (FBS) environments. The retention of these signals in T‐Ag_SA_‐CDs, but not in N‐CDs or Ag_SA_‐CDs, indicates that the functionalization is chemically stable and not merely due to physical adsorption. This structural integration of the T molecule probably makes the nanozyme more redox active and biocompatible. The zeta potentials of N‐CDs, Ag_SA_‐CDs, and T‐Ag_SA_‐CDs in a pH 7.4 environment were −17.18, −25.80, and −6.4, respectively (Figure S9c). In addition, we examined the effect of H_2_O_2_ on the nanoparticle morphology (Figure S10). Under H_2_O_2_‐stimulated conditions, the particle size and shape remained unchanged, with no observable signs of leaching or agglomeration. T molecules are neutral organophosphorus compounds that facilitate interaction with Ag_SA_‐CDs via coordination or weak intermolecular forces, promoting adequate mixing and adsorption.

### 2.2 Structural and Chemical Characterization of Ag_SA_‐CDs

**FIGURE 2:**
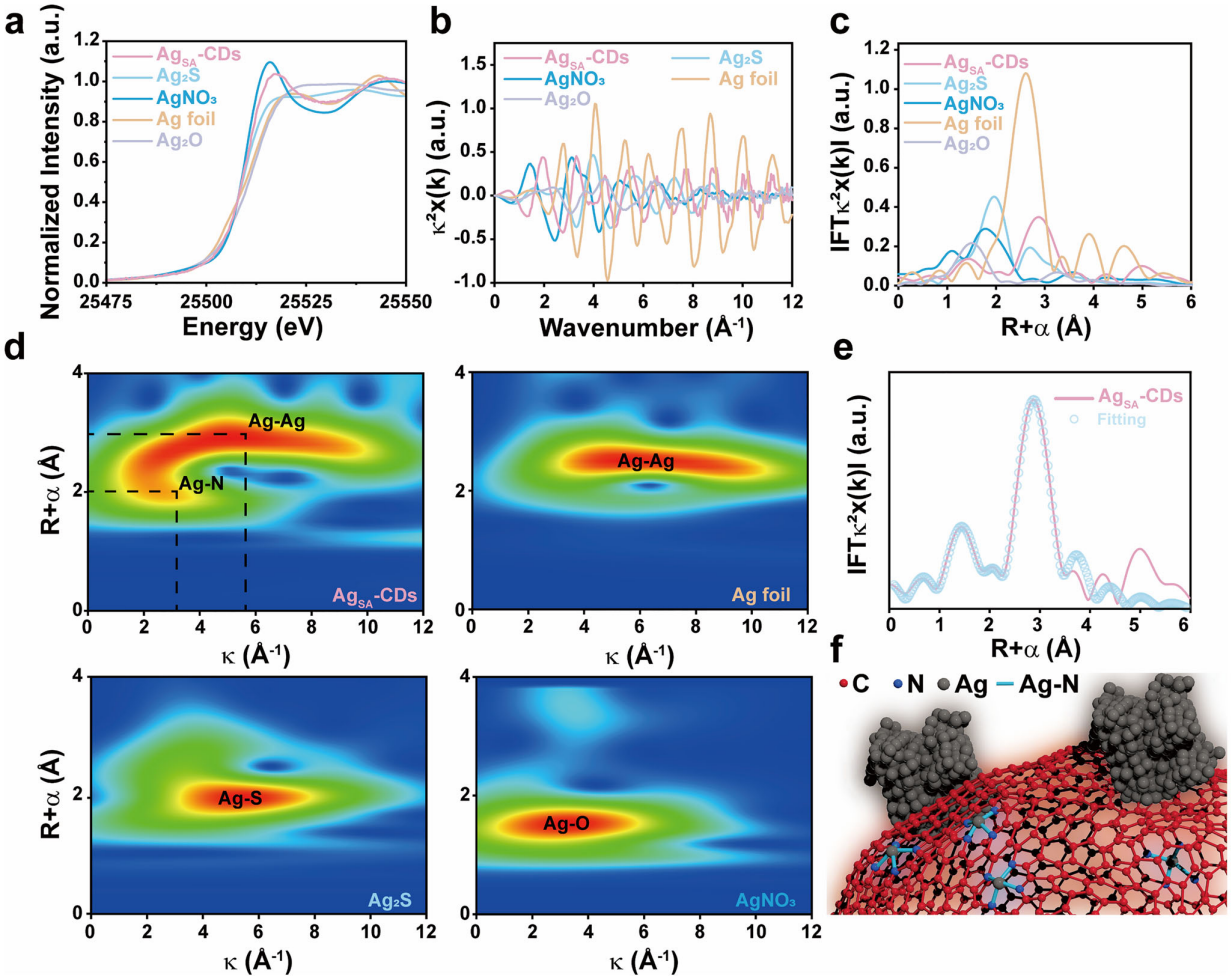
Atomic structural characterization of Ag_SA_‐CDs nanozyme. a) XANES spectra of Ag K‐edge in Ag_SA_‐CDs nanozyme. b) k^2^‐weighted Fourier transforms EXAFS spectra of Ag_SA_‐CDs, Ag foil, Ag_2_O, AgNO_3_, and Ag_2_S samples. c) Fourier transform of Ag K‐edge EXAFS spectra of Ag_SA_‐CDs nanozyme, Ag foil, Ag_2_O, AgNO_3_, and Ag_2_S samples. d) Wavelet transforms (WT) contour plots of the k^3^‐weighted EXAFS data of Ag_SA_‐CDs and reference samples (Ag foil, AgNO_3_, and Ag_2_S). e) The corresponding EXAFS fitting curves of Ag_SA_‐CDs ‐nanozyme at r space. f) a model surface structure of Ag_SA_‐CDs nanozyme.

The X‐ray absorption spectroscopy (XAS) including the X‐ray absorption near‐edge structure (XANES) and extended X‐ray absorption fine structure (EXAFS) has been carried out to investigate the local chemical environment and electronic state of individual Ag atoms in the Ag_SA_‐CDs nanozymes. To contextualize the coordination environment of Ag_SA_‐CDs, we made a comprehensively comparison with some benchmark materials, including metallic such as Ag foil (metallic Ag^0^), Ag_2_O (Ag^+^), AgNO_3_ (Ag^+^), and Ag_2_S (Ag^+^). The XANES profile of Ag_SA_‐CDs, as illustrated in Figure 2a, reveals a near‐edge absorption energy that falls between that of Ag foil and Ag_2_O, suggesting a partially oxidized valence state of Ag. Consequently, Ag in Ag_SA_‐CDs exists in a unique electronic environment, which distinguishes it from both bulk metallic and fully oxidized Ag species. Besides, a strong peak at 25510 eV from XANES spectrum of Ag_SA_‐CDs indicates specific feature relevant to the planar Ag‐Ag or Ag‐N type coordination sites points to a single‐atom structure. The local atomic structure was further confirmed by the EXAFS analysis, which reveals that Ag atoms in Ag_SA_‐CDs are mainly coordinated by N or C atoms and also form large‐sized quantum molecular state (QMS) metallic clusters or Ag─Ag bonds just like seen for Ag foil (Figure 2b). The typical oscillation of the EXAFS spectra does not include Ag‐N/C coordination pertaining to reference compounds. The Ag_SA_‐CDs induce a slight increase in the Ag valence state through an interaction between Ag and N/C atoms, as confirmed by XANES edge shift. This transfer indicates the redistribution of charge around the single atom Ag, prompted by electron donation from N or C ligands that stabilize the single‐atom species.

The Fourier transform EXAFS results (Figure 2c) show that the peaks at ≈2.40 and ≈2.84 Å correspond to Ag─N(C) and Ag─Ag bonds, respectively, which further indicates that Ag exists in the form of single atoms and cluster shape. The EXAFS data‐fitting results suggested that the coordination number of Ag‐N(C) and Ag─Ag in the Ag_SA_‐CDs was 10.3 ± 2.1 and 10.7 ± 2.1, respectively (Figure 2d; Table S3). Furthermore, the wavelet transformation contour plots based on the EXAFS spectra show that Ag‐N_4_ and Ag─Ag scattering paths were present in the Ag_SA_‐CDs (Figure 2e). In other words, one Ag atom combines with three N or C or Ag atoms to form the atomically dispersed Ag‐N_4_ or Ag‐Ag structural unit. Based on the above characterizations, a possible structural diagram of Ag_SA_‐CDs is provided (Figure 2f). Thus, the XAS and XPS data work clasping each other's hands, revealing that Ag_SA_‐CDs are made up of single‐atom Ag and clusters integrated into N‐CDs. This material boasts a highly graphitic structure, with Ag^0^ evenly distributed and closely coordinated with N and C, resulting in exceptional catalytic performance.

### 2.3 Enzyme‐Mimic Properties of N‐CDs/Ag_SA_‐CDs Nanozymes

**FIGURE 3:**
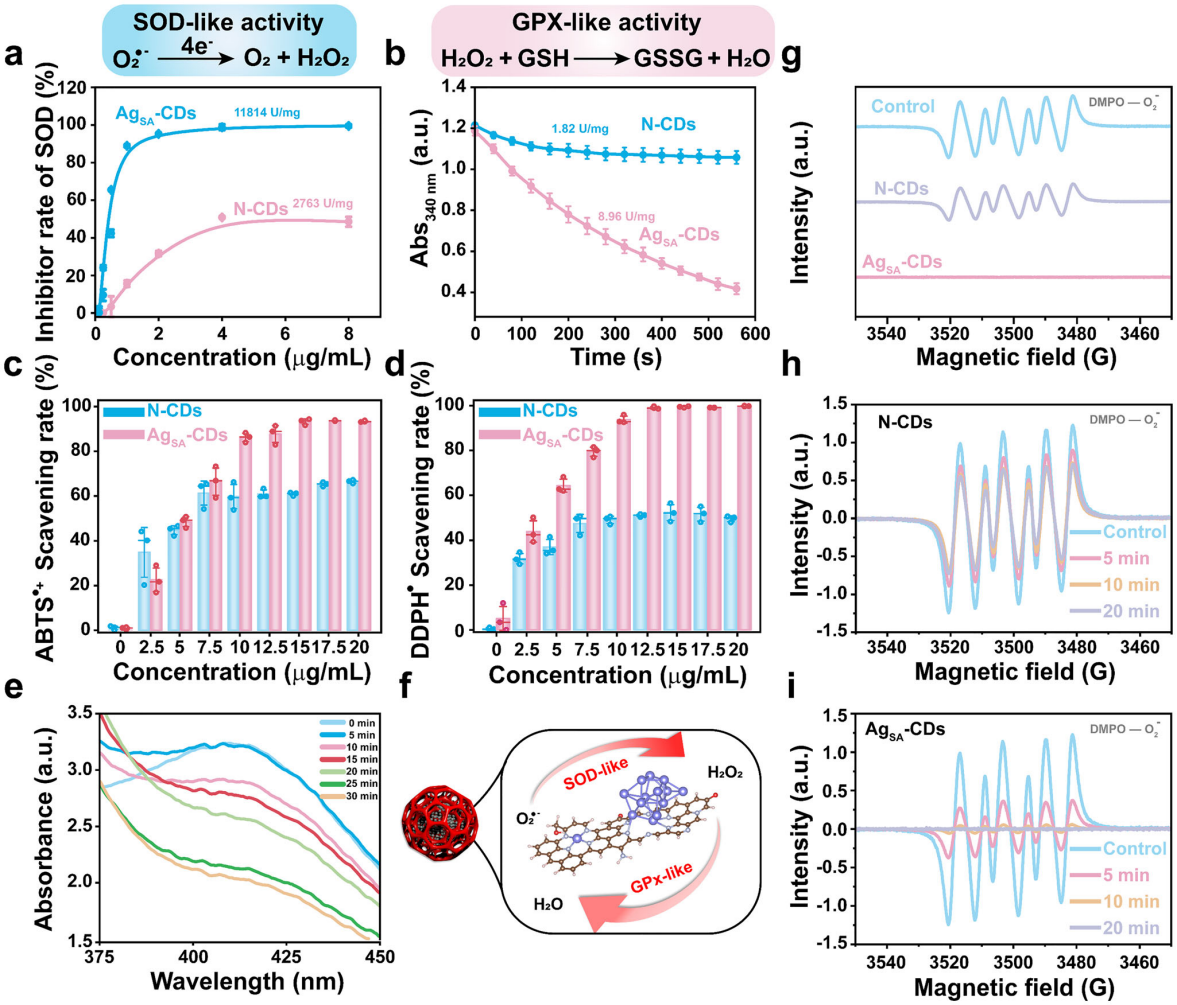
Enzyme kinetics of Ag_SA_‐CDs and N‐CDs nanozymes. Kinetics for SOD‐like (a), and GPx‐like (b) activities of Ag_SA_‐CDs and N‐CDs nanozymes. (c–e), Comparative studies of ABTS^•+^ (c), and DPPH• (d) and GSH trapping (e) scavenging ability of Ag_SA_‐CDs and N‐CDs. In (c,e), data represent means ± s.d. from 4 independent replicates. g) O_2_•^−^ scavenging activities of Ag_SA_‐CDs and N‐CDs nanozymes. Time‐dependent ESR adduct of O_2_•^−^ scavenging activities of N‐CDs (h), and Ag_SA_‐CDs (i), respectively.

In this study, we quantitatively measured the SOD‐like and GPX‐like activities of N‐CDs or Ag_SA_‐CDs nanozymes with clarity and precision. SOD enzymes (or their mimics) catalyze the dismutation of O_2_•^−^ radicals into molecular oxygen (O_2_) and H_2_O_2_, as depicted in the reaction scheme at the top (Figure 3a). The above process serves mitigate oxidative stress by neutralizing one of the primary ROS in biological systems. Figure 3a, a dose‐response curve plot showing the percentage (%) inhibition of an O_2_•^−^ radical source generated Ag_SA_‐CDs/N‐CDs as a function of nanozyme concentration (0–8 µg/mL). At low concentrations (e.g., 2 µg/mL), inhibition is modest (∼20%–40%), but it rises sharply with increasing dose, approaching ∼98% at 8 µg/mL. The Ag_SA_‐CDs nanozyme consistently outperforms the N‐CDs nanozyme across all concentrations, achieving ∼98% inhibition by 8 µg/mL compared to ∼50% for N‐CDs. This suggests that Ag doping enhances the catalytic efficiency, possibly by modulating surface electronic properties or active sites that facilitate O_2_•^−^ binding and electron transfer. The maximum SOD‐like activities of 11 814 and 2 763 U/mg for Ag_SA_‐CDs and N‐CDs nanozyme, respectively, which shows ∼4.3‐fold higher activity of Ag_SA_‐CDs than N‐CDs, highlighting the benefit of Ag‐doping for ROS scavenging applications. Moreover, the Ag_SA_‐CDs surpass the natural bovine Cu/Zn‐SOD that serves as a benchmark, many inorganic and CDs‐based SOD nanozymes (Table S4). Thus, the Ag‐doping provides a clear edge, likely due to Ag facilitating O_2_•^−^ adsorption and reduction (similar to Cu in natural SOD) [37].

GPx is a vital member of the peroxidase enzyme family and helps regulate H_2_O_2_ levels in cells. This enzyme uses GSH, a small cellular antioxidant, to transform H_2_O_2_ into harmless water (H_2_O), while converting GSH into its oxidized form (GSSG). The process entails the conversion of GSSG to GSH, which is catalyzed by the enzyme GSH reductase (GR) in conjunction with the coenzyme nicotinamide adenine dinucleotide phosphate (NADPH), as shown in the equation given in Figure 3b. The GPx‐like activity of Ag_SA_‐CDs and N‐CDs nanozymes was evaluated by monitoring the real‐time reduction in NADPH signal, as illustrated in Figure 3b. A rapid drop in NADPH absorbance over time suggests that Ag_SA_‐CDs effectively mimic GPx activity. In contrast, a lower limit to no GPx‐like activity was observed in N‐CDs under identical conditions. To investigate the GPx‐like activity of Ag_SA_‐CDs, we performed Michaelis‐Menten dynamic equilibrium kinetic studies utilizing H_2_O_2_ as the substrate (Figure S11). The findings indicated Km and Vmax values of 0.091 mM and 12.02 µ M/min, respectively, for Ag_SA_‐CDs (see Table S5), emphasizing their strong catalytic potential. Simultaneously, we also assayed the peroxidase (POD)‐like activity for N‐CDs and Ag_SA_‐CDs using TMB oxidation by classical Michaelis–Menten kinetic analysis (Figure S12). Compared to the Km value of N‐CDs (57.51 µM), the Ag_SA_‐CDs have a much lower Km (406.75 µm), which indicates that Ag_SA_‐CDs exhibited 7–8 times lower substrate affinity, with 4 times higher Vmax of 0.024 µm s^−^^1^. The markedly lower Km and higher catalytic turnover depict that atomically dispersed Ag–N_4_ sites are effectively maintained for TMB binding and electron transfer.

After proving the antioxidant nanozyme properties of the Ag_SA_‐CDs and N‐CDs, we then studied their ability to neutralize ROS in physiological conditions, specifically to scavenge •OH and other free radicals effectively. We assessed antioxidant properties using 2,2′‐azino‐bis(3‐ethylbenzothiazoline‐6‐sulfonic acid) (ABTS•^+^) and 1,1‐diphenyl‐2‐picrylhydrazyl (DPPH•) radical scavenging assays, with dose‐response curves for vigorous and broad‐spectrum antioxidant activity. As shown in Figure 3c,d, with increasing concentrations of Ag_SA_‐CDs and N‐CDs, the ABTS and DPPH radicals were effectively scavenged, and the absorbance gradually decreased. At 5 µg/mL, both catalysts had nearly the same scavenging rate; as the concentration increased, the rate surpassed 90%. In this comparative analysis, the Ag_SA_‐CDs nanozyme shows greater ABTS and DPPH radicals than N‐CDs. We next examined the capacity of Ag_SA_‐CDs to scavenge H_2_O_2_ in the presence of GSH, which promotes the reduction of oxidized GSSG to its reduced form (GSH). The antioxidant activity of Ag_SA_‐CDs was evaluated using 5,5'‐dithiobis (2‐nitrobenzoic acid) (DTNB) as a thiol‐reactive molecular probe to measure the transformation levels from GSH to GSSG. After a 30‐min incubation with AgSA‐CDs, we observed a time‐dependent reduction in the absorbance at 412 nm from DTNB, which reflects GSH consumption due to increased H_2_O_2_ scavenging (Figure 3e). The findings from the DTNB assay indicated that Ag_SA_‐CDs alleviate the decomposition of H_2_O_2_, probably by the synergistic interaction with GSH, which successively helps in the reduction of GSSG.

**FIGURE 4:**
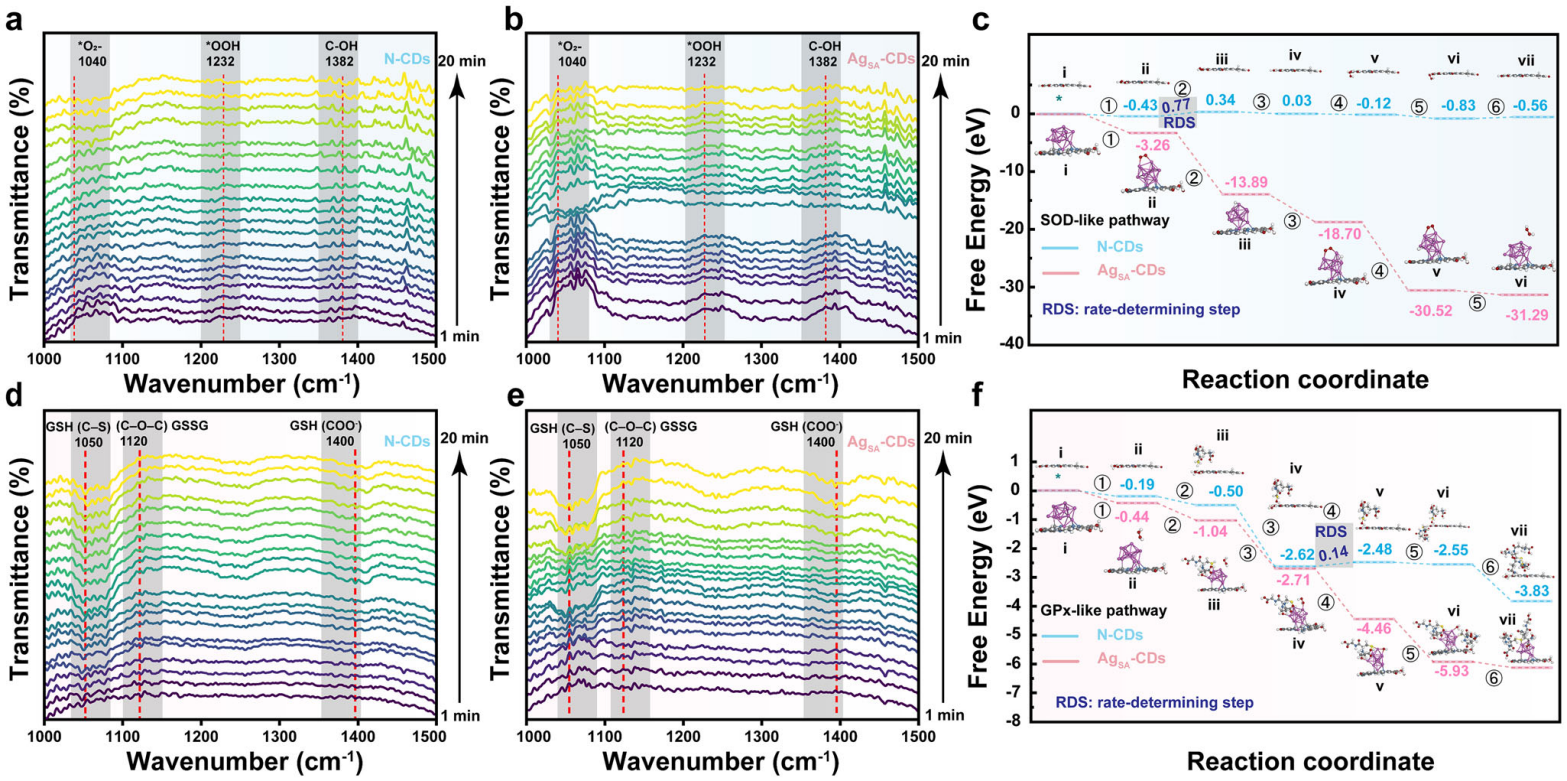
In situ DRIFTS spectra of ROS and GSH transformation pathways on N‐CDs and Ag_SA_–CDs. (a,b) Time‐resolved signatures of O_2_− (1040 cm^−1^), OOH (1232 cm^−1^), and surface C─OH (1382 cm^−1^) during O_2_/H_2_O_2_ activation on N‐CDs and Ag_SA_–CDs nanzoyme. (d,e) Evolution of GSH and GSSG characteristic bands of C─S (1050 cm^−1^), C─O─C (1120 cm^−1^); COO(1400 cm^−1^) showing catalytic GSH oxidation. c, f) Reaction energy barrier diagram of potential pathways profiles for ROS scavenging and the catalytic pathways on N‐CDs and Ag_SA_‐CDs nanozymes. c) SOD‐like pathway for N‐CDs and Ag_SA_‐CDs reflecting enhanced superoxide dismutation efficiency. f) GPx‐like pathway for N‐CDs and Ag_SA_‐CDs indicating improved H_2_O_2_ reduction.

To validate the SOD‐like activities and ROS scavenging capabilities of N‐CDs/ Ag_SA_‐CDs nanozymes in further detail, we employed electron spin resonance (ESR) spectroscopy, an accurate and specific method for detecting O_2_•^−^ by utilizing 5,5‐dimethyl‐1‐pyrroline N‐oxide (DMPO) as the spin‐trapping reagent. Figure 3g illustrates that the ESR signal intensity of the DMPO/•OOH (DMPO‐O_2_•^−^) spin adduct was significantly diminished in the presence of Ag_SA_‐CDs nanozyme compared to N‐CDs and the control group under identical conditions. The reduced signal intensity seen with Ag_SA_‐CDs suggests improved O_2_•^−^ scavenging, which can be linked to the exceptional catalytic properties of the Ag factor in the nanozyme. When we compared the time‐dependent ESR spin adduct of DMPO/•OOH, it strongly supported the fact that Ag_SA_‐CDs (Figure 3i) nanozymes have significantly reduced the production of O_2_•^−^ compared with the N‐CDs counterpart (Figure 3h). Interestingly, the time‐dependent measurements (control, 5, 10, and 20 min) show that the spin adducts of N‐CDs tend to a progressive increase in the DMPO‐O_2_•^−^ signal intensity over time, reaching a peak at 20 min (Figure 3h). Thus, it indicates that ROS generation by N‐CDs is time‐dependent, with a gradual accumulation of O_2_•^−^ radicals. Contrary to the N‐CDs data, the Ag_SA_‐CDs reinforce the superior catalytic activity, which is attributed to the synergistic effect of silver and CDs. Further information about enzyme‐mimicking properties can be found in mechanistic insights from DFT calculations in Figure 4. Thus, these data show that the outstanding antioxidant properties make the Ag_SA_‐CDs boost the biomedical application, especially to work for ROS‐related diseases.

### 2.4 Experimental and Theoretical Analysis of the Mechanism and Energetics Derived From DFT

To study the reaction mechanism of N‐CDs and Ag_SA_‐CDs nanozymes, time‐resolved in situ diffuse reflectance infrared Fourier transform spectroscopy (DRIFTS) was employed to monitor the evolution of H_2_O_2_ dismutation (SOD‐like activity) and the consumption of H_2_O_2_‐dependent thiol oxidation (GPx‐like activity). Figure 4a shows that the peaks at 1040 and 1232 cm^−1^ represent the v(O−O) stretching mode of O_2_•^−^ and ^*^OOH species, respectively. The peak at 1232 cm^−1^ trend indicates that O_2_•^−^ is being proportionally transformed into •OOH and other peroxyl intermediates. The rising intensity shows the catalytic pathway in which Ag_SA_‐CDs help move electrons and proton transfer steps, turning superoxide into hydroperoxide species in a way that corresponds to SOD‐like activity. The signal at 1382 cm^−1^ was ascribed to Ag‐coordinated superoxo/peroxo and potentially Ag–OOH intermediates. Pristine N‐CDs, SOD‐like (Figure 4b), triggered minimal spectral changes over 10 min. Weak features at 1382 and 1040 cm^−^^1^ peaks regions indicated no considerable development of O_2_‐derived intermediates on carbon‐based active sites. In agreement with our DFT findings, breaking and forming O─O bonds have considerable activation barriers (RDS >0.77 eV) and tightly bonded superoxo/peroxo species develop on pyridinic/graphitic N centers. A delocalized radical or outer‐sphere electron‐transfer mechanism with minimal spectroscopic imprint is compatible with proving our hypothesis. However, Ag_SA_‐CDs showed considerable time‐dependent spectrum fluctuations under the same circumstances. In SOD‐like H_2_O_2_ dismutation, a significant band at 1382, 1040, and a peak at 1227 cm^−^^1^ were ascribed to various intermediate steps of Ag to *OOH species. We found that all these peaks reached their equilibrium state within 5 min and then gradually started dropping with time preceding. The vibration at 1382 cm^−^^1^ is comparable to metal‐coordinated superoxo (O_2_^−^) or hydroperoxo (OOH^−^) species on silver (reported 1350–1400 cm^−^^3^ for Ag─O_2_^−^ and Ag─OOH complexes).

In the GPx‐like cycle (Figure 4d,e), a strong band at ∼1380–1390 cm^−^^1^ formed immediately and persisted. Likewise, the peak at 1051 cm^−1^ was assigned to C─S stretching in reduced GSH, which decreases increasingly with time, pointing to continuous thiol oxidation. Additionally, Ag─O and Ag─SS─O stretching modes caused extensive absorptions in the 1250–1350 cm^−^^1^ region. As time extended, the peak at 1120 cm^−1^, which is attributed to C─O─C stretching of the oxidized glutathione (GSSG), was gradually intensified. The increase of the COO band at ∼1400 cm^−^^1^ indicates the deprotonation and structural rearrangements during the thiol‐to‐disulfide (R‐S‐S‐OH) conversion, which is consistent with basal GPx‐like activity. The species in question are identical to the intermediates (Ag─O_2_^−^, Ag^3^^+^ = O, and Ag─OOH) observed in our DFT‐optimized catalytic cycles on the Ag_SA_ center. The Ag location makes activating the O─O bond (0.14 eV) and switching between O_2_ and thiol intermediates is easier. The in‐situ DRIFTS results demonstrate that Ag_SA_‐CDs use a conventional inner‐sphere, metal‐centered redox process with transitory high‐valent silver–oxo (Ag─O_2_^−^) and silver–peroxo (Ag─OOH) species. In contrast, N‐CDs use a carbon‐dominated pathway with few surface‐bound intermediates.

After confirmation of time‐resolved spectroscopic validation of various intermediates, the particular SOD‐ and GPx‐like activities of T‐Ag_SA_‐CDs stem from their ability to overcome thermodynamic bottlenecks in ROS scavenging, as elucidated by density functional theory (DFT) free‐energy profiles. These profiles reveal that incorporating single‐atom Ag into Ag_SA_‐CDs fundamentally enhances the catalytic efficiency for O_2_•^−^ dismutation and H_2_O_2_ reduction compared to metal‐free N‐CDs. All the proposed pathways and the structures of the intermediates in the two models for SOD‐like and GPx‐like pathways over N‐CD and Ag_SA_‐CDs nanozymes (Figure S13 and the corresponding complete reaction schemes for the SOD‐ and GPx‐like pathways are provided in Supporting Information). In view of the fact that experiments, the proposed models were designed to represent the mildly basic environment that favors H_2_O_2_ production in the SOD‐like pathway and the physiological pH that supports H_2_O_2_ consumption in the GPx‐like cycle. In the N‐CD model (Figure 4c), the SOD‐like sequence exhibits a moderate endergonic step recognized as the rate‐determining step (RDS, approximately +0.77 eV), linked to the formation and transformation of the *OOH/* OOH/* OOH/* OOH/* OOH/*O‐containing intermediates. After this RDS, the path goes downhill, but the positive (+eV) free‐energy barrier shows that there is a thermodynamic bottleneck that will slow down turnover. In contrast, the Ag_SA_‐CD profile (Figure 4c) stabilizes adsorbed O_2_, and the reduced O intermediates are much more strongly exergonic. This behavior is consistent with the Ag center functioning as a localized electron reservoir and adsorption site that (i) facilitates O_2_ adsorption and partial reduction to O_2_^−^/*O_2_ species, (ii) diminishes the O─O bond through back‐donation/charge transfer, and (iii) stabilizes *OOH/*O intermediates. The overall effect is a considerable drop in the thermodynamic cost of the PCET (proton‐coupled electron transfer) events needed to change O_2_•^−^, which means that SOD‐like activity on Ag_SA_‐CDs happens faster.

A comparable trend is noted for the GPx‐like H_2_O_2_ to convert H_2_O, facilitated by GSH. In the case of N‐CDs (Figure 4f), there are several exergonic steps, but there is still a shaded RDS (RDS: 0.14) region. The significant energetic barrier to H‐transfer or the formation of GS‐bound intermediates was observed. On the other hand, Ag_SA_‐CDs (Figure 4f) exhibited a substantially more favorable energy landscape, in which each elementary step, including H‐transfer to H_2_O_2_, formation of *OH or *OOH intermediates, and the eventual conversion to GSSG formation, is markedly stabilized by several electron volts compared to the metal‐free surface. Thereby, a single atom Ag promotes either heterolytic or homolytic activation of H_2_O_2_ and reduces the free‐energy barrier for the crucial H‐atom/proton transfer from GSH to H_2_O_2_. In other words, Ag creates an active site that links substrate adsorption with easy PCET events needed for GPx‐like turnover. The observed energetic enhancements can be explained employing traditional single‐atom catalyst principles. The Ag center modifies the local density of states and charge distribution, resulting in enhanced, albeit potentially transient, interactions with oxygenated intermediates. N‐doping of the carbon scaffold probably changes the oxidation state of the Ag and arranges it in a way that balances how strongly it adsorbs and how reactive it is. Insets in the panels that show adsorbate geometries are consistent with an Ag‐centered adsorption motif (rather than delocalized physisorption on the carbon surface), which explains the considerable stabilization of intermediates.

The Ag nanoclusters in Ag_SA_‐CDs have been secondary electron intermediary structures, rather than catalytic centers of action. The experimental ROS‐scavenging kinetics and DFT‐derived reaction energetics indicated that the isolated Ag–N_x_ single‐atom sites govern the SOD‐like and GPx‐like pathways. At the same time, the nanoclusters modulate the carbon framework's electronic environment, accelerating the Ag_SA_ site through combining the redox cycling and stabilizing transient reaction intermediates. This cooperative effect supports that collectively driven charge transfer between atomic and nanometer Ag species boosts multienzyme activity [24]. Thus, Ag nanoclusters may indirectly affect catalytic efficiency through stimulating electron‐transfer dynamics; however, Ag single atoms determine the catalytic mechanism.

Taken together, the in situ DRIFT measurements and the DFT analyses provide a coherent description of the catalytic behavior. The incorporation of single‐atom Ag transforms the N‐CDs from moderately active surfaces into highly efficient nanozyme centers capable of stabilizing and converting both O_2_•^−^ and H_2_O_2_ intermediates and GSH/GSSG transformations. This dual improvement in SOD‐like and GPx‐dependent H_2_O_2_ pathways explains the experimentally observed improvements in ROS scavenging and mitochondrial protection integrity. These findings highlight an essential design principle: embedding isolated metal atoms within heteroatom‐enriched carbon frameworks creates well‐defined sites that promote O_2_•^−^ and H_2_O_2_ activation through charge transfer while reducing PCET barriers for both O_2_•^−^ and GSH‐dependent H_2_O_2_ reduction.

### 2.5 Cytoprotective and Antioxidant Activity Effects of Ag_SA_‐CDs and N‐CDs Nanozymes

**FIGURE 5:**
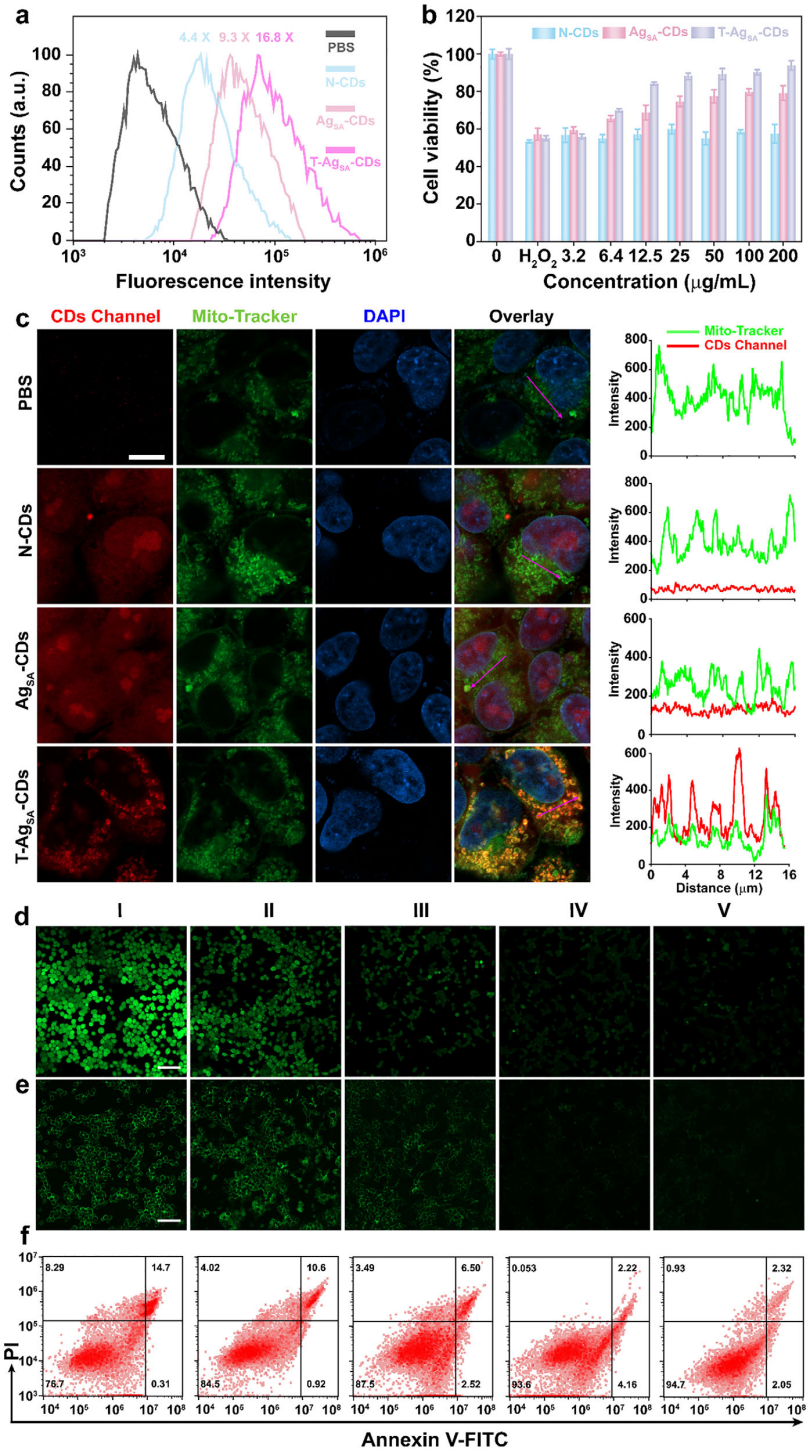
Assessment of N‐CDs, Ag_SA_‐CDs, and T‐Ag_SA_‐CDs nanozymes in HEK293T kidney cells. a) Intracellular uptake of Ag_SA_‐CDs and N‐CDs nanozymes in 293T cells after 6 h incubation by flow cytometry analysis. b) Effect of N‐CDs, Ag_SA_‐CDs, and T‐Ag_SA_‐CDs on cell viability against 293T cells under H_2_O_2_‐stressed conditions (*n* = 3). c) Confocal images of the co‐localization between N‐CDs, Ag_SA_‐CDs, and T‐Ag_SA_‐CDs and mitochondria in 293T cells (mitochondria stained by Mitotracker (green), nuclei stained by DAPI (blue), red‐emissive CDs (red), (Scale bar: 10 µm). d) CLSM imaging of intracellular ROS using DCFH‐DA probe (Scale bar: 100 µm). e); CLSM imaging of mitochondrial ROS using mitoSOX green probe (Scale bar: 100 µm). f). Flow cytometric analysis of apoptosis in 293T cells using Annexin V‐FITC/PI dual staining. Cells were exposed to a 250 µM H_2_O_2_ challenge prior to a 12‐h treatment with the indicated CDs (50 µg/mL). (I: H_2_O_2;_ II: H_2_O_2_ + N‐CDs; III: H_2_O_2_ + Ag_SA_‐CDs; IV: H_2_O_2_ + T‐Ag_SA_‐CDs; V: PBS).

The onset of the cytoprotective effect was observed following the uptake of Ag_SA_‐CDs and N‐CDs nanozymes in HEK293T cells. Figure 5a shows the flow cytometric histogram overlays describing the fluorescence intensity distributions for untreated PBS control (black) and cells treated with N‐CDs (blue), Ag_SA_‐CDs (pink), and T‐Ag_SA_‐CDs (red) nanozymes uptake in HEK293T cells. Interestingly, the T‐Ag_SA_‐CDs provided a considerable accumulation of 16.8x time higher than the control group and other treatment groups. A progressive rightward shift in fluorescence peaks indicates devastating internalization, with T‐Ag_SA_‐CDs exhibiting the highest uptake efficiency. In addition, before conducting in vitro biological evaluations, it is essential to assess the cytotoxicity of Ag_SA_‐CDs and N‐CDs nanozymes in the HEK293T cell line. After exposing the cells to various concentrations of N‐CDs, Ag_SA_‐CDs, and T‐Ag_SA_‐CDs nanozymes for 24 h, we found no significant cytotoxicity, either with (Figure 5b) or without (Figure S14) H_2_O_2_, suggesting that these nanozymes are well‐tolerated in vitro. Additionally, given the potential toxicity linked to the Ag^+^ ion, we investigated the Ag^+^ release profile of Ag_SA_‐CDs under acidic conditions (lysosomal pH 5.0), mitochondrial pH (6.5), and physiological conditions, along with medium‐dependent behavior over a 72‐h duration (Figure S15). In acidic buffer (pH 4.5), an initial rapid release of approximately 0.45 µg/mL was observed at 24 h, succeeded by a sustained release, suggesting proton‐induced destabilization of Ag‐thiolate coordination. At physiological pH levels of 6.5 and 7.5, we observed a significant inhibition of release, with measurements less than 0.25 µg/mL. Complex media (DMEM and DMEM+10% FBS) demonstrated minimal Ag^+^ release (<0.1 µg/mL) consistently, attributable to robust binding interactions with amino acids and serum proteins that efficiently sequester free Ag^+^ ions, which is supported by our previous study [38]. The results demonstrate the remarkable stability of Ag_SA_‐CDs in physiological conditions, with release occurring exclusively in acidic microenvironments.

We further investigated the cellular penetrability of Ag_SA_‐CDs and N‐CDs nanozymes because of their robust fluorescent characteristics (Scheme S1). After incubation with N‐CDs, Ag_SA_‐CDs, and T‐Ag_SA_‐CDs (20 µg mL^−1^) in HEK293T cells, analyzed by flow cytometry, which suggests that Ag_SA_‐CDs and N‐CDs nanozymes may remain stable within the intracellular milieu, essential for the continuous imaging and modulation of ROS levels in living cells. Then, we tested the subcellular colocalization of CDs in HEK293T cells. Lysosomes are mainly responsible for phagocytosis of foreign substances, while mitochondria are key for producing O_2_•^−^, which contributes to oxidative stress within the cell. Hence, the localization of CDs was investigated in these essential organelles. HEK293T cells were incubated with N‐CDs, Ag_SA_‐CDs, and T‐Ag_SA_‐CDs (red channel), followed by vibrant staining of lysosomes and mitochondria using LysoTracker green and MitoTracker green, respectively, to visualize their intracellular dynamics. As clearly shown in Figure 5c and Figure S16 fluorescence imaging, the N‐CDs, Ag_SA_‐CDs, and T‐Ag_SA_‐CDs were colocalized with mitochondria thanks to the targeting moiety (with T‐Ag_SA_‐CDs), achieving superior mitochondrial targeting due to the cell‐penetrating T molecule, which contributed to the enhancement of the oxidative capacity of the Ag_SA_‐CDs nanozyme. However, as the nanozyme works in physiological conditions, it restricted its penetration and colocalization within the acidic microenvironment (pH 4–5) of the lysosome (Figure S16). Therefore, these data suggest targeted mitochondrial delivery of Ag_SA_‐CDs over the lysosome that obeys the endo/lysosome escape path, which was clearly indicated in intensity profiles (right panels) across various groups.

We conducted further investigations using confocal laser fluorescence microscopy to study the cytoprotective effects of CDs against H_2_O_2_‐induced oxidative stress. Our findings shed light on the role of CDs in protecting cells from oxidative damage, suggesting potential therapeutic applications. Thus, the ROS‐scavenging capacity of N‐CDs, Ag_SA_‐CDs, and T‐Ag_SA_‐CDs was consistently investigated using the ROS‐specific green emission fluorescent probe (DCFH‐DA), which oxidizes into fluorescent DCF in ROS‐induced by H_2_O_2_ stressed conditions. Figure 5d and Figure S17a,b demonstrated that HEK293T cells stimulated with H_2_O_2_ exhibited enhanced green fluorescence signals signifying elevated intracellular ROS levels. However, after treatment of the oxidative stress‐generated HEK293T cells with N‐CDs, Ag_SA_‐CDs, and T‐Ag_SA_‐CDs, we observed that the levels of ROS decreased to levels comparable to those in the control group, providing sufficient effectiveness in ROS neutralization, owing to the supplying of robust antioxidant cytoprotection.

We also examined mitochondrial ROS generation employing MitoSOX green staining, a selective marker for mitochondrial superoxide. Confocal imaging and flow cytometry data (Figure 5e and Figure S18a,b) strongly correlate with H_2_O_2_ treatment, which increases mitochondrial O_2_•^−^ production (15.6%). However, after T‐Ag_SA_‐CDs treatment, this value was significantly decreased, providing substantial evidence of tremendous protective impact. Thereby, these results strongly indicate the possibility of Ag_SA_‐CDs nanozyme as a targeted therapeutic agent to elicit oxidative stress.

The higher redox stress led to a series of events that resulted in disturbed cellular homeostasis. As we noticed, the great ROS‐scavenging ability of Ag_SA_‐CDs and T‐Ag_SA_‐CDs contributes to alleviating oxidative stress and disturbance in mitochondrial function. Their ability to counteract ROS could potentially prohibit the oxidative stress‐induced hyperpolarization of the mitochondrial membrane potential (ΔΨm), promoting cellular health, as presented in Figure S19. The fluorescence imaging of JC‐1 dye revealed dramatic hyperpolarization and depolarization of mitochondria (green) after treatment with CCCP, H_2_O_2_, and H_2_O_2_ plus N‐CDs. Moreover, the intact ∆Ψm (red color) induced by interactive H_2_O_2_ after co‐incubating with N‐CDs, Ag_SA_‐CDs, and T‐Ag_SA_‐CDs was also interesting when compared to the PBS control group. The results provide substantive evidence that N and Ag doping inhibit the apoptotic inducer of HEK293T cells, resulting in JC‐1 monomer generation in damaged cells with low ∆Ψm, and that T‐Ag_SA_‐CDs exert protective effects against cells insulted by oxidative stress. The findings further demonstrated that T‐Ag_SA_‐CDs provide adequate protection to cells against damage induced by oxidative stress.

To investigate the protective effects of N‐CDs and Ag_SA_‐CDs, we added them to HEK293T cells with H_2_O_2_ induction to examine whether they could lower the degree of oxidative stress induced by H_2_O_2_. In particular, we examined how these nanozymes influence the inhibition of the apoptotic cascade, revealing their potential to mitigate cell death under oxidative stress conditions (Figure 5f). The T‐Ag_SA_‐CDs show > 20‐fold decrease in apoptosis compared to the stressed control (H_2_O_2_, denoted as I) and demonstrate a superior performance over N‐CDs (∼6‐fold) and Ag_SA_‐CDs (∼8‐fold). The effectiveness of the nanozyme comes from its ability to mimic both GPX and SOD enzymes. The core‐shell N‐CDs structure together with Ag doping plays a synergistic role in the effective dismutation of toxic/microbicidal H_2_O_2_ and O_2_•^−^ radicals, helping to accelerate the electron transfer rate and enhancing antioxidant behavior. The conjugation of this T molecule induces ROS neutralization in a spatiotemporal manner that is necessary for maintaining ATP homeostasis. These results provide further support for the in vitro protective role of our nanozyme systems in potential theranostic applications. The cellular responses to various treatments were further observed by BioTEM data (Figure S20). We witnessed the significant cellular damage caused by H_2_O_2_ and H_2_O_2_ + N‐CDs groups, with disrupted membranes and increased presence of dark spots indicating oxidative stress and cell injury. However, the cellular damage was recovered by treatment with Ag_SA_‐CDs, with more intact cellular structures and fewer autophagosomes. These findings demonstrate that T‐Ag_SA_‐CDs offer better protection than H_2_O_2_ for the cells that are oxidatively stressed.

To estimate the intracellular antioxidant capacity of CDs‐based nanozymes, cellular ATP level, GPX activity, and lactate dehydrogenase (LDH) release were measured in H_2_O_2_‐induced oxidative‐stressed cells. Consistently, as shown in Figure S21a, H_2_O_2_ treatment reduced intracellular ATP levels markedly, indicating strong mitochondrial dysfunction. Meanwhile, cellular ATP levels were partially recovered by N‐CDs and Ag_SA_‐CDs but also significantly improved by T‐Ag_SA_‐CDs to the level that nearly approached physiological conditions in the PBS‐treated group. Similarly, GPX activity, an important antioxidant defense enzymatic system, was significantly decreased after exposure to H_2_O_2_ in Figure S21b. N‐CDs and Ag_SA_​‐CDs moderately restored GPX activity, while T‐Ag_SA_‐CDs exhibited superior efficacy, demonstrating activity comparable to the PBS group, thereby indicating potent intracellular ROS scavenging capability.

On the other hand, the H_2_O_2_ group exhibited a significantly higher degree of LDH leakage than the control, indicating damage to the cell membrane (Figure S21c. N‐CDs and Ag_SA_‐CDs reduced LDH leakage by a moderate amount, while T‐Ag_SA_‐CDs significantly reduced LDH release, bringing levels close to those of the PBS group. Taken together, these results demonstrate that CDs‐based nanozymes with T‐Ag_SA_‐CDs can effectively alleviate the oxidative stress‐related cellular injury. It further suggests that the protection of its mitochondrial function is not only a surrogate marker, but represents mainly the target of ROS‐induced damage, as it leads to restoration of intracellular ATP levels. The enhanced GPX‐like capacity suggests that these nanozymes not only directly scavenge ROS but may also cooperate with the endogenous antioxidant system to balance the redox state. The significant reduction of LDH leakage on their part reflects the ability to maintain intracellular membrane integrity in oxidizing conditions. Overall, these findings strongly support that newly developed CDs‐based nanozymes are promising antioxidant agents with potential applications in the management of oxidative stress‐related diseases, including AKI.

Inflammation plays a key role in the development of AKI and ROS‐related diseases, and the crosstalk between renal tubular epithelial cells and immune cells affects tissue repair and functional recovery, which is quite complex [39]. We monitored the anti‐inflammatory effects of N‐CDs, Ag_SA_‐CDs, and T‐Ag_SA_‐CDs by measuring the amount of representative proinflammatory cytokines induced in H_2_O_2_‐treated HEK293T cells. The effect of H_2_O_2_ on the pro‐inflammatory cytokines (TNF‐α and IL‐6) response was also highly stimulated in cells, as shown in Figure S22. The activities of the N‐CDs, Ag_SA_‐CDs, and T‐Ag_SA_‐CDs had the potential to improve this tendency, indicating a strong anti‐inflammatory effect. In the current study, the T‐Ag_SA_‐CDs caused a remarkable suppression of TNF‐α and IL‐6 expression as compared to N‐CD and non‐modified Ag_SA_‐CDs, which indicated that there may be synergistic inhibition of inflammation after introducing these components.

### 2.6 Molecular Modulation of Nanozyme Triggered Various ROS‐Mediated Pathways

**FIGURE 6:**
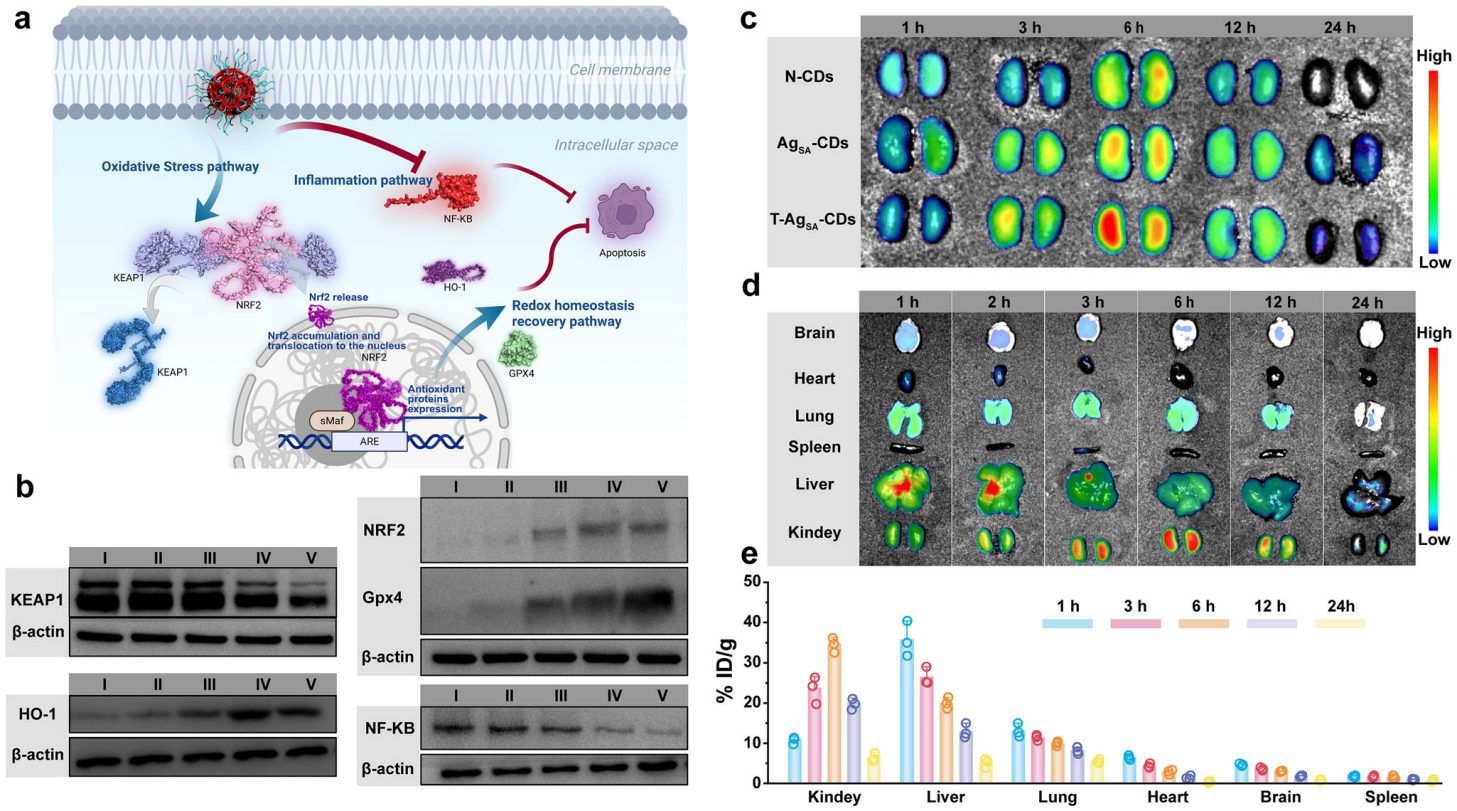
Molecular and cellular responses to CDs based AKI response. a) Schematic representation of the cellular pathway involving KEAP1, NRF2, and downstream targets (HO‐1, GPX4) in response to AKI. b) Western blot analysis showing protein expression levels of KEAP1, NRF2, GPX4, HO‐1, and NF‐κB across experimental conditions (I: H_2_O_2_; II: H_2_O_2_ + N‐CDs; III: H_2_O_2_ + Ag_SA_‐CDs; IV: H_2_O_2_ + T‐Ag_SA_‐CDs and V: PBS) against β‐actin as a loading control. c) Ex vivo fluorescence imaging of N‐CDs, Ag_SA_‐CDs, and T‐Ag_SA_‐CDs at 1, 3, 6, 12, and 24 h, indicating time‐dependent changes in uptake and fluorescence intensity. d) Ex vivo biodistribution data of CDs in brain, heart, lung, spleen, liver, and kidney at 1, 2, 3, 6, 12, and 24 h, visualized through fluorescence imaging with a color scale representing signal intensity. e) Quantitative analysis of T‐Ag_SA_‐CDs accumulation (% ID/g) in major organs (kidney, liver, lung, heart, brain, and spleen) time points of post‐injection (*n* = 3 biologically independent mice). The i.v. dosage: 200 µL. Error bars represent standard deviations (*n* = 3).

The protective effects of Ag_SA_‐CDs were additionally investigated against oxidative stress in AKI pathology with an emphasis on cellular mechanisms within the kidney and biodistribution after in vivo administration. As depicted in Figure 6a, a diagram demonstrates the therapeutic mechanisms of T‐Ag_SA_‐CDs nanozyme—entering renal tubular and tubulous cells by ROS‐generating stressors. In the pathological state, excessive ROS oxidizes and inactivates Kelch‐like ECH‐associated protein 1 (KEAP1), resulting in detachment of KEAP from nuclear factor erythroid 2‐related factor 2 (NRF2), nuclear localized NRF2, and subsequent transcriptional activation of antioxidant genes including heme oxygenase‐1 (HO‐1) and glutathione peroxidase 4 (GPX4). However, unimpeded ROS also initiates the activity of nuclear factor kappa B (NF‐κB), which leads to proinflammatory signaling and apoptosis. T‐Ag_SA_‐CDs nanozyme constructed by Ag_SA_‐modified N‐CDs showed SOD‐like and GPx‐like activities for scavenging O_2_•^−^ and H_2_O_2_, which can restore KEAP1‐NRF2 equilibrium, upregulate HO‐1 and GPX4 expression, inhibit NF‐κB activation, and finally alleviate apoptosis.

As followed by a series of western blot analysis (Figure 6b) confirms the molecular modulation in H_2_O_2_‐stimulated human HEK293T cells, a standard in vitro model of AKI. Lane I‐V comprises the H_2_O_2_, H_2_O_2_ + N‐CDs, H_2_O_2_ + Ag_SA_‐CDs, H_2_O_2_ + T‐Ag_SA_‐CDs and PBS, respectively. KEAP1 expression is upregulated significantly in both H_2_O_2_ and N‐CDs groups; intensity was normalized to β‐actin, representing oxidative damage inactivation of NRF2 release. However, the treatment of T‐Ag_SA_‐CDs (lane V) shows a remarkable decrease of KEAP1, much lower than N‐CDs or Ag_SA_‐CDs alone, suggesting synergistic disassembly between wild‐type KEAP1 and NRF2. In contrast, H_2_O_2_ exposure downregulates NRF2 and HO‐1 levels, although these can be reinstated to the point of near‐mimic control values by T‐Ag_SA_‐CDs. GPX4 has a similar trend; we find that H_2_O_2_‐induced depletion is restored by the nanozyme, indicating its role as a ferroptosis inhibitor. It has been reported that NF‐κB p65 subunit is increased under stress and decreased by T‐Ag_SA_‐CDs, indicating the presence of an anti‐inflammatory effect. β‐actin serves as a loading control across blots. The KEAP1‐NRF2 system is a key rheostat that maintains cellular redox homeostasis, especially in the kidney, which is vulnerable to oxidative stress in AKI owing to its high energy demand. In the basal state, KEAP1 functions as an adaptor protein for Cullin‐3/RING‐box‐1 E3 ubiquitin ligase‐dependent proteasomal degradation of NRF2 to maintain low steady‐state levels of NRF2. Pathological ROS levels, as mimicked in this study with H_2_O_2_ or cisplatin, covalently modify cysteine residues (e.g., Cys151, Cys 273/288) within KEAP1 that lead to conformational changes, releasing NRF2 for translocation into the nucleus and forming heterodimers with small Maf proteins. These data drives antioxidant response element (ARE)‐dependent transcription of cytoprotective genes like HO‐1 and GPX4 (which reduces phospholipid hydroperoxides to prevent ferroptotic lipid peroxidation). However, in AKI, this activation is often transient and insufficient, as evidenced by the observed KEAP1 hyper‐expression and NRF2/HO‐1/GPX4 downregulation in untreated cells (Figure 6b, lane I), leading to NF‐κB hyperactivation. NF‐κB translocation promotes pro‐inflammatory cytokine release (e.g., TNF‐α, IL‐1β) and caspase‐3‐mediated apoptosis, exacerbating tubular necrosis and renal dysfunction. To this end, the T‐Ag_SA_‐CDs nanozyme overcomes this discrepancy at its best by taking advantage of catalysis biomimicking, utilizing single‐atom Ag coordination in the N‐CDs structure to imitate the action of an enzyme. The incorporated N dopants can increase the electron density at carbon edges, which stabilizes Ag atoms with pseudo‐square‐planar N_4_ coordination (Ag‐N_4_C) and thus reduces the activation energy of O_2_•^−^ dismutation (like‐standard Cu/Zn‐SOD) and successive H_2_O_2_ decomposition (mimicking CAT or GPx). This self‐cascading process (better than N‐CDs or Ag_SA_‐CDs alone, Figure 6b) could continuously scavenge ROS for up to 24 h and prevent KEAP1 from being oxidized, thereby achieving long‐duration NRF2 activation. As a result, the upregulation of HO‐1 and GPx4 (Figure 6b, lane IV) not only eliminates residual ROS but also prevents NF‐κB p65 phosphorylation on Ser536, uncoupling oxidative stress from inflammation at this key juncture in the progression of AKI to CKD.

After observing that T‐Ag_SA_‐CDs elicited a potent antioxidant cellular response, we next monitored the CDs in mice with or without AKI models. According to Figure 6c, the behavior of N‐CDs, Ag_SA_‐CDs, and T‐Ag_SA_‐CDs can be monitored by the time‐dependent uptake response and ROS‐scavenging ability of nanozymes in the case of AKI. N‐CDs exhibit rapid internalization within 1 h, appearing as fluorescent puncta, with sustained intracellular distribution up to 24 h. Ag_SA_‐CD shows similar kinetics but with enhanced brightness and fluorescence intensity, attributed to Ag‐mediated fluorescence enhancement. T‐Ag_SA_‐CDs display the most efficient uptake, with diffuse cytosolic and perinuclear localization by 3–6 h, correlating with peak ROS quenching (color scale: high green intensity indicates low residual ROS). By 12–24 h, T‐Ag_SA_‐CDs maintain low ROS levels, outperforming individual components, which supports their multienzyme‐mimetic cascade (SOD‐like O_2_•^−^ dismutation to H_2_O_2_, followed by GPX‐like decomposition). In vivo biodistribution was further visualized in Figure 6d using intravital fluorescence imaging in a cisplatin‐induced AKI mouse model at post‐injection time points (1–24 h). T‐Ag_SA_‐CDs preferentially accumulate in the kidney, showing intense fluorescence signals in the renal cortex and medulla by 3 h, peaking at 6–12 h, and persisting modestly at 24 h. However, the low uptake was observed in the brain, heart, lung, liver, and spleen, indicating renal tropism likely due to glomerular filtration and tubular reabsorption of the ultrasmall less than 10 nm) nanozyme. This selectivity contrasts with the broader distribution of free Ag_SA_‐CDs or N‐CDs (Figure 6c), underscoring the role of the CDs matrix's particularity in size‐ and surface‐dependent targeting chemistry. We further performed the quantitative biodistribution data in Figure 6e, measured via fluorescence intensity for T‐Ag_SA_‐CDs content (% injected dose per gram tissue,% ID/g), which corroborates the bio‐imaging data. The accumulation of the CDs in the kidney reaches ∼45% ID/g at 1 h, declining to ∼20% ID/g by 24 h, with rapid clearance (<5% ID/g in off‐target organs). Transient maximum values are also detected in the liver (∼10%–15% ID/g at 3 h) and spleen, indicative of reticuloendothelial system uptake, whereas lung, heart, and brain do not go beyond 5% ID/g over the evaluation time.

The hemocompatibility of the T‐Ag_SA_‐CDs nanozyme was systematically evaluated by measuring the hemolysis ratio of red blood cells (RBCs) at various concentrations ranging from 0 to 1000 µg mL^−^^1^. As shown in Figure S23, all tested concentrations of T‐Ag_SA_‐CDs exhibited negligible hemolytic activity, with the hemolysis ratio remaining below 3%, even at the highest concentration of 1000 µg mL^−^^1^. In contrast, the positive control (dd H_2_O) induced complete hemolysis, resulting in a deep red supernatant due to the release of hemoglobin. The inset photograph clearly demonstrates the absence of red coloration in the T‐Ag_SA_‐CDs‐treated blood, confirming that the nanozyme does not disrupt the integrity of RBC membranes. The apparent color shown in the vial originated from the CDs, as it is redder in color as a red‐emissive fluorescent probe. In addition, these findings indicate that T‐Ag_SA_‐CDs possess excellent blood compatibility, as no observable hemolytic response was detected across the physiologically relevant concentration range. For pharmacokinetic studies, N‐CDs, Ag_SA_‐CDs, and T‐Ag_SA_‐CDs were administered to the healthy mice via i.v. tail injection. Fluorescence intensity (Ex/Em = 560/650 nm) was recorded at different post‐injection time intervals. Notably, T‐Ag_SA_‐CDs have a prolonged plasma elimination half‐life (t_1/2_, β) of 119.1 min, while un‐modified Ag_SA_‐CDs and N‐CDs were recorded as 69.91 and 44.73 min, respectively (Figure S24). These data proved that ligand T bioconjugation to CDs improves the pharmacokinetics of nanozymes in plasma.

These results affirm the nanozyme's favorable pharmacokinetics for AKI therapy, minimizing systemic exposure. Overall, these results coincide with recent findings that nanozyme‐mediated ROS regulation is more effective than small‐molecule antioxidants in AKI because it provides long‐lasting, site‐specific catalysis without denaturing the enzyme. T‐Ag_SA_‐CDs is an exceptional example that has shown >11,000 U/mg SOD‐like activity along with decomposition of H_2_O_2_ in the form of GPX‐like activity in different oxidative models. They are more stable than natural enzymes at pH 5–8 and temperatures up to 60°C. In the context of AKI, the augmentation of NRF2 independent of KEAP1 inhibition has been associated with decreased tubular fibrosis. These findings indicate that T‐Ag_SA_‐CDs may offer benefits beyond acute protection, potentially preventing chronic progression.

### 2.7 In Vivo Therapeutic Assessments of T‐Ag_SA_‐CDs for AKI

**FIGURE 7:**
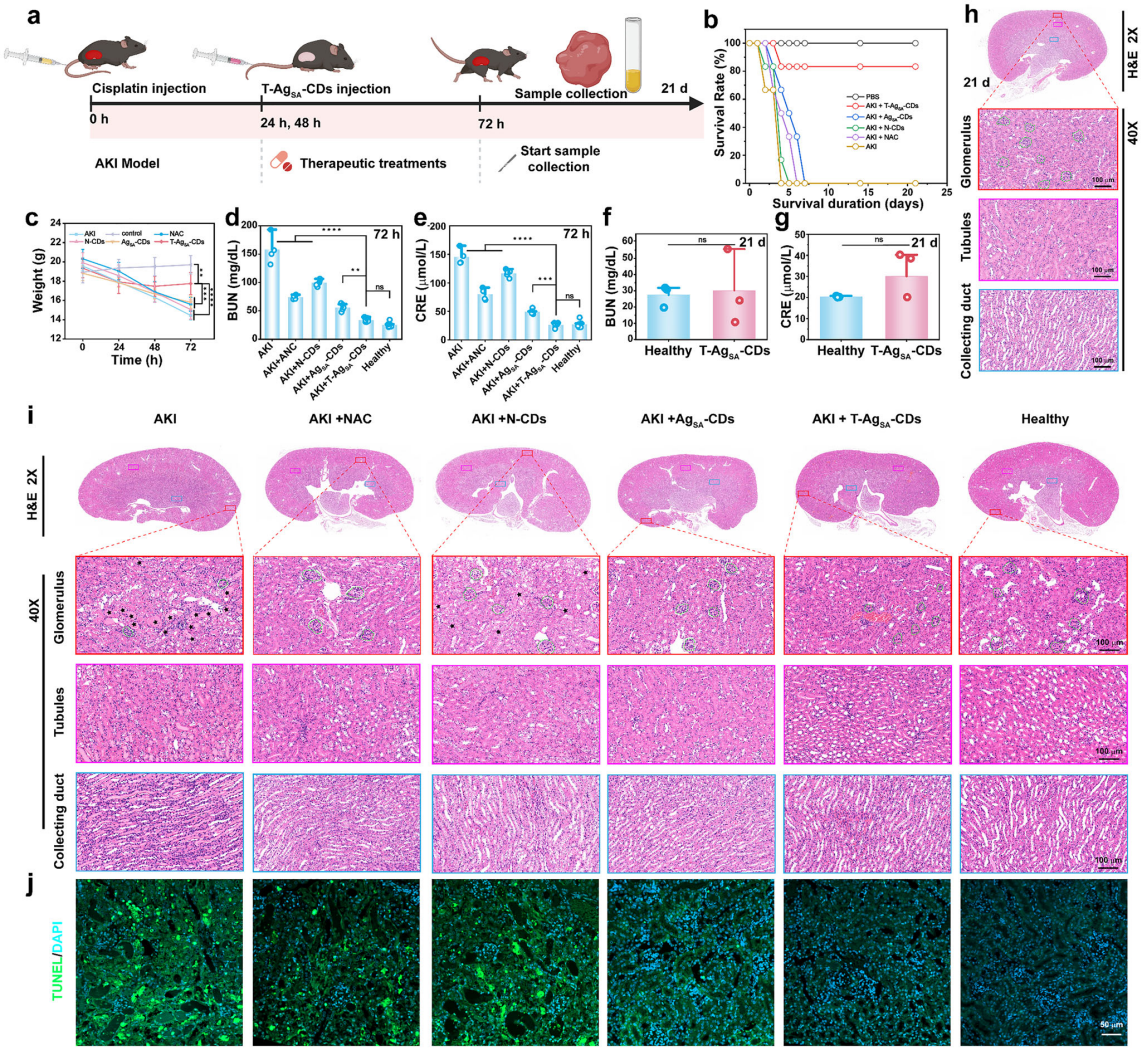
Therapeutic efficacy of T‐Ag_SA_‐CDs in cisplatin‐induced AKI. (a) Schematic illustration of the experimental protocol: AKI was induced by cisplatin injection (0 h), followed by administration of T‐Ag_SA_‐CDs or control treatments (24 and 48 h), with functional and histological post cure assessments performed at 72 h and 21 d. (b) Survival analysis of AKI mice treated with saline, NAC, N‐CDs, Ag_SA_‐CDs, or T‐Ag_SA_‐CDs (*n* = 6 per group). (c) Body weight variation over 72 h following treatment. (d,e) BUN and serum CRE levels at 72 h showing significantly improved renal function in the treated groups. (f,g) Long‐term renal function biomarkers (BUN and CRE) on day 21, demonstrating sustained functional recovery and post cure assessment after T‐Ag_SA_‐CDs treatment. h) Representative H&E‐stained kidney sections from healthy and T‐Ag_SA_‐CDs–treated mice at 21 d showing preserved histoarchitecture at the whole‐organ (2×) and cellular levels (40×; glomerulus, tubules, collecting duct). i) Comparative H&E images of kidneys from AKI mice treated with NAC, N‐CDs, Ag_SA_‐CDs, or T‐Ag_SA_‐CDs at 72 h, highlighting the superior protection of glomeruli, tubules, and collecting ducts in the T‐Ag_SA_‐CDs group relative to extensive injury in untreated AKI. j) TUNEL/DAPI staining of renal tissue sections demonstrating reduced apoptotic cell death in T‐Ag_SA_‐CDs–treated kidneys compared with other treatment groups. Scale bars: 100 µm (H&E 40×), 50 µm (TUNEL assay). (i.v. dosage: 1 mg/mL (10 mg/kg)of N‐CDs, Ag_SA_‐CDs, or T‐Ag_SA_‐CDs and NAC). Data are presented as mean SD (*n* = 6, one‐way ANOVA and Tukey multiple comparisons tests, ^*^ *p *< 0.05, ^***^ *p *< 0.001, ^****^ *p *< 0.0001).

Inspired by the promising ability of Ag_SA_‐CDs nanozyme for ROS clearance in vitro, along with its favorable circulation, targeting, and biodistribution in AKI mice, we further investigated its therapeutic potential for treating ROS‐related AKI in mice. Figure 7a provides an overview of the modeling and therapy of mouse AKI in a continuum. The AKI model was established through i.v. injection, and the cisplatin‐mediated AKI model followed by nanozyme administration with longitudinal monitoring. After 24, 48, and 72 h, all the mice were sacrificed, and renal function test and H&E staining were conducted for the therapeutic assessments (Figure S25a). The significantly elevated levels of serum creatinine (CRE) and blood urea nitrogen (BUN) in AKI mice compared with the control group suggested that renal dysfunction was pronounced (Figure S25c,d). The weight changes also suggested that the mice were more likely to be at risk (Figure S25b). Hence, the drug‐induced AKI model was successfully established and ready for therapeutic actions.

The therapeutic effect of the T‐Ag_SA_‐CDs nanozyme in AKI was comprehensively evaluated by in‐vivo experiment, and functional recovery, as well as histological preservation, was proved. Most notably, the survival duration of T‐Ag_SA_‐CDs‐treated mice was significantly prolonged compared to N‐acetylcysteine (NAC), N‐CD, or non‐targeted Ag_SA_‐CDs‐treated ones, indicating a synergistic action of Ag_SA_ atoms combined with renal‐targeted CDs. The physiological parameters corroborated the therapeutic efficacy. Weight loss is indicative of systemic toxicity and was significantly reduced by T‐Ag_SA_‐CDs treatment (Figure 7c). Concurrently, renal function biomarkers such as BUN and CRE demonstrated significant reduction at 72 h in the T‐Ag_SA_‐CDs group relative to other treatments (Figure 7d,e). The post‐cure assessment is one of the urgent demands for modern time therapeutics. The protective effects of nanozyme were maintained until day 21, corresponding with ongoing renal recovery (Figure 7f,g). Interestingly, the mice treated with T‐Ag_SA_‐CDs survived for 21 days, and their BUN and CRE level recovered to almost those of the healthy control group. Biochemical (BUN, creatinine) and histological (H&E) analyses on day 21 were performed only on surviving animals, as shown previously in Figure 7b. Since the T‐Ag_SA_‐CDs group had a significantly higher survival rate than the other AKI therapy groups (Figure 7b), we only have endpoint data for healthy controls and mice treated with T‐Ag_SA_‐CDs. The maintenance of long‐term renal function indicates both acute protection from oxidative stress and the prevention of progressive kidney damage, which is critical for translating nanozyme therapies to clinical settings. Histological and immunofluorescence examinations validated the translating nanozyme therapies' results. Hematoxylin and eosin (H&E) staining at both 72 h and 21 days indicated that T‐Ag_SA_‐CDs efficiently maintained glomerular and tubular architecture, exhibiting minimal necrosis or dilatation compared to the significant structural damage seen in untreated AKI mice (Figure 7h,i). Similarly, the post‐cure assessment data were compared for mice with AKI treated with T‐Ag_SA_‐CDs to healthy controls using H&E examination (Figure 7h, T‐Ag_SA_‐CDs‐treated H&E tissues data versus healthy subject Figure S26). It is important to note that NAC and N‐CDs only offered partial protection, and their effects were not as strong as those of T‐Ag_SA_‐CDs. In this way, it shows how important it is to have accurate structural design and renal targeting to get the best results from a treatment. Additionally, TUNEL (Figure 7j), an immunofluorescence staining, also indicated that apoptosis was significantly decreased in T‐Ag_SA_‐CDs–treated kidneys, confirming the function of the nanozyme on attenuating oxidative‐induced apoptosis in pathological conditions of cisplatin damage.

In addition, in order to gain a deeper insight into the molecular mechanisms of T‐Ag_SA_‐CDs mediated protective effects against cisplatin‐induced AKI, different protein levels of essential oxidative stress‐associated signaling indicators were analyzed by immunofluorescence staining and quantification (Figure S27a,b). The findings demonstrated marked stimulation of endogenous antioxidant pathways in T‐Ag_SA_‐CDs, when compared with untreated AKI and both control and untargeted groups. The major regulator of redox homeostasis, Nrf2, was highly upregulated in T‐Ag_SA_‐CDs‐treated kidneys and was strongly localized to the nucleus via enhancement indicative of transcriptional activation. By contrast, Nrf2 expression was barely observed in untreated AKI, and moderate upregulation of Nrf2 in the therapy of NAC, N‐CDs, or non‐targeted Ag_SA_‐CDs was also observed. Expressions of the Nrf2 axis downstream molecules, including HO‐1 and GPX‐4, were also dramatically up‐regulated in T‐Ag_SA_‐CDs. These data suggest that the nanozyme scavenges ROS not only directly via catalytic Ag single atoms but also promotes endogenous cytoprotective signaling. The dual mechanism of catalytic ROS neutralization and reinforcement of host antioxidant defenses likely explains the significant functional and histological protection observed in previous analyses. Ag_SA_‐CDs induced partial activation of these markers; however, the extent was significantly less than that observed with T‐Ag_SA_‐CDs. This highlights the critical role of renal‐targeting ligands in facilitating adequate intrarenal accumulation and pathway modulation. NAC and N‐CDs, while traditionally acknowledged as antioxidants, did not significantly activate the Nrf2/HO‐1/GPX‐4 axis, indicating their limitations in bioavailability and catalytic efficacy relative to atomically dispersed Ag‐atom systems. The immunofluorescent investigation is strongly correlated with our derived cellular mechanism that is triggered by T‐Ag_SA_‐CDs nanozyme.

Collectively, these results demonstrate that the therapeutic effects of T‐Ag_SA_‐CDs are attributed to the co‐contribution of three factors: (i) atomically dispersed Ag atoms promoting high catalytic efficiency to scavenge ROS with GPX‐ and SOD‐like activities; (ii) mitochondria‐targeting unit enabling selective accumulation in lesioned kidney tissues; and (iii) robust CDs backbone endowing biocompatibility and prolong circulation time. This multi‐interaction mode distinguishes T‐Ag_SA_‐CDs from common antioxidant NAC, which has low bioavailability and limited effectiveness.

These findings show that T‐Ag_SA_‐CDs are a potent and durable strategy to treat cisplatin‐induced AKI, which provides a basis for future studies on the utilization of designed targeted SAzyme therapy in addressing oxidative stress‐mediated organ injuries. The mechanistic evidence demonstrates that T‐Ag_SA_‐CDs exert their therapeutic efficiency not only due to the ROS scavenging potential but are also involved in upregulation of endogenous antioxidative and anti‐apoptotic signaling pathways. The synergistic modulation is essential in AKI, where unmanaged oxidative stress leads to both necrotic and apoptotic cell death. The data in aggregate highlight the potential benefit of a selectively engineered organ‐specific single‐atom nanozyme to modulate intracellular stress pathways, thus representing a shift from passive ROS scavengers to active inducers of cytoprotective signaling.

### 2.8 Biosafety Evaluation of Ag_SA_‐CDs

**FIGURE 8:**
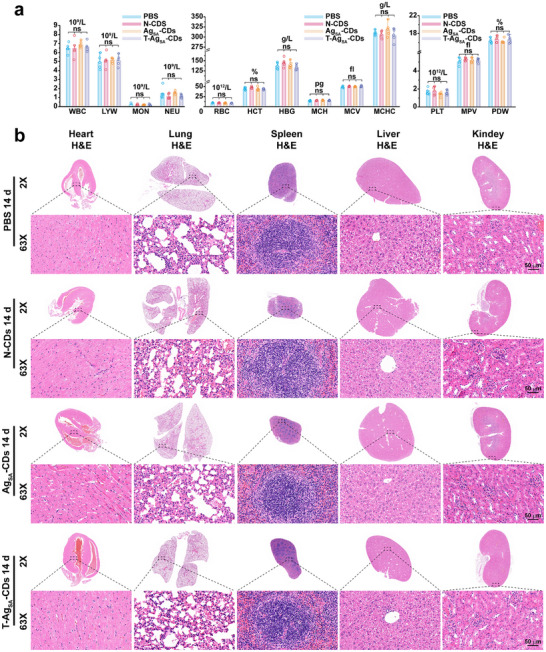
Hematological and histopathological evaluation of the biosafety of T‐Ag_SA_‐CDs. (a) Hematology parameters of mice 14 days after administration of PBS, N‐CDs, Ag_SA_‐CDs, or T‐Ag_SA_‐CDs, including white blood cell counts (WBC, LYW, MON, NEU), red blood cell indices (RBC, HCT, HBG, MCV, MCH, MCHC), and platelet parameters (PLT, MPV, PDW), showing no significant deviations across groups. (b) Representative H&E‐stained sections of major organs (heart, lung, spleen, liver, and kidney) from each treatment group at 14 days. Images at whole‐organ (2×) and magnified (63×) levels reveal no evidence of structural damage, necrosis, or inflammatory infiltration, confirming the systemic biosafety of the formulations. Scale bars: 200 µm (2×), 50 µm (63×).

The safety of nanotherapeutics is a primary challenge in clinical translation, and the therapeutic effect must be accompanied by an extensive hematological and histopathological analysis. Therefore, we conducted extensive evaluation and post‐cure investigation of the in vivo safety aspects of T‐Ag_SA_‐CDs in relation to N‐CDs and Ag_SA_‐CDs after 14 days (Figure 8). CBC readouts revealed that there were no statistical differences in leukocyte, erythrocyte, hemoglobin, or platelet counts in all treatment groups compared with PBS controls (Figure 8a). The stability of these subsets (LYW, MON, and NEU) suggests the nanozymes did not cause observable immunostimulation or immunosuppression. The indices related to RBC (RBC, HCT, HBG, MCV, MCH, and MCHC) and platelets (PLT, MPV, and PDW) are also within the physiological range of each group, with no indication of hematotoxicity or ability to maintain their blood production (compared to the control). The findings indicate that systemic exposure to T‐Ag_SA_‐CDs does not disrupt major hematological homeostasis.

Histopathological analysis confirmed the biosafety of the nanozyme formulations (Figure 8b). H&E staining revealed no evidence of necrosis, inflammation, perforation, or structural damage in all major vital organs, including the heart, lung, spleen, liver, and kidney, in the T‐Ag_SA_‐CDs–treated group, at both whole‐organ (2×) and tissue‐magnified (63×) levels. As clearly shown, the myocardium retained its characteristic myofibrillar arrangement, the lung alveolar architecture showed no alterations due to infiltration or edema, the splenic white pulp displayed a well‐organized structure, the hepatic lobules maintained their regular sinusoidal formations, and the renal cortex and medulla preserved their typical glomerular and tubular morphology. Both the N‐CDs and Ag_SA_‐CDs groups had comparable safety and no definite signs of organ damage. The lack of cumulative or delayed organ toxicity at 14 days demonstrates that the carbon dot scaffold offers biocompatibility while facilitating the stability and dispersibility of atomically distributed Ag. The importance of these findings is rooted in the combination of therapeutic efficacy and biosafety. Systemic toxicity problems have restricted traditional Ag‐based nanomaterials [40]. Nevertheless, atomically dispersed Ag bound to N‐CDs is only weakly beneficial for improved safety by preventing aggregation and uncontrolled release of Ag^+^ ions as well as specific accumulation, such as targeted in the diseased tissue. The lack of hematological disturbance or histological abnormality from T‐Ag_SA_‐CDs suggests that the fabricated T‐Ag_SA_‐CDs are catalytically effective and systemically safe to remove oxidative stress in AKI.

## 3 Conclusion

In summary, we presented T‐Ag_SA_‐CDs as an innovative theranostic nanozyme for the treatment of AKI. T‐Ag_SA_‐CDs exhibit exceptional SOD‐ and GPx‐like activities by utilizing the distinctive characteristics of Ag_SA_ and nanoclusters within a CDs matrix, resulting in greater ROS scavenging efficiency relative to traditional nanozymes. Experimental validation and DFT calculations have shown the low‐energy reaction pathways that make their dual‐enzyme mimetic abilities possible. In addition, the biocompatible CDs platform enhances stability and reduces toxicity to Ag^+^ ions while simultaneously improving catalytic performance by facilitating appropriate molecular interactions. The T ligand functionalization allows for targeted mitochondrial ROS neutralization, which dramatically improves the results of treatment in a mouse model of AKI caused by cisplatin. Moreover, the inherent fluorescence of T‐Ag_SA_‐CDs enables real‐time, non‐invasive bioimaging, facilitating accurate observation of biodistribution, renal accumulation, and therapeutic advancement. T‐Ag_SA_‐CDs represent a significant advancement in nanomedicine by integrating diagnostic imaging with multi‐enzyme mimetic functions. Importantly, the drug induced AKI was fully restored after the T‐Ag_SA_‐CDs treatment after 21 days, which lays the groundwork for the advancement of next‐generation theranostic platforms, presenting promising prospects for the treatment of AKI and other ROS‐mediated diseases.

## 4 Experimental Section

### 4.1 Materials

All chemicals and reagents used in this study were of analytical grade and sourced from specified suppliers unless otherwise noted. Citrate, urea, potassium persulfate (K_2_S_2_O₈), Xanthine (X755591), Xanthine Oxidase (B3SPS5), Glutathione (Reduced) (G105426), DMSO (D670381), NHS, (4‐Carboxybutyl)triphenylphosphonium bromide (TPP‐COOH), 2,2′‐Azino‐bis(3‐ethylbenzothiazoline‐6‐sulfonic acid) diammonium salt (ABTS) were purchased from Aladdin (Shanghai, China). Formic acid, silver nitrate (AgNO_3_), Sodium borohydride (NaBH_4_), Trisodium citrate dihydrate were obtained from Xilong Scientific (China). The SOD assay kit was purchased from Dojindo (Japan). Glutathione Peroxidase (GPX) Assay Kit, Annexin V‐FITC Apoptosis Detection Kit, mitochondrial membrane potential assay kit with JC‐1, and LDH (Lactate Dehydrogenase) were purchased from Beyotime Biotechnology. DAPI(4',6‐diamidino‐2‐phenylindole), Mito‐tracker green, Lyso‐tracker green, and Mitosox‐green were purchased from ThermoFisher. N‐(3‐Dimethylaminopropyl)‐N′‐ethylcarbodiimide hydrochloride was obtained from Macklin. 1,1‐diphenyl‐2‐picrylhydrazyl (DPPH) and 2’‐7’dichlorofluorescin diacetate (DCFH‐DA) were obtained from MedChemExpress (MCE). Cell counting kit‐8 (CCK‐8), ATP Assay Kit, and Marker (10∼180 KDa) were purchased from Biosharp (China). The antibodies used in this study were supplied by Proteintech (China), unless otherwise noted in the relevant method section. High‐glucose Dulbecco's Modified Eagle Medium (DMEM), streptomycin‐penicillin, and non‐essential amino acids were obtained from Thermo Fisher Scientific (Suzhou). Fetal Bovine Serum was sourced from Procell Life Science & Technology Co., Ltd. All experiments used deionized water purified by a Milli‐Q system (Merck, Germany), with a resistivity of −18mῼ/cm^2^. All flasks used in the cell culture experiments were obtained from Nest Biotechnologies Co., Ltd. (Wuxi, Jiangsu, China). We sourced HEK293T cells (RRID: CVCL_0063) from Procell Life Science Technology Co., Ltd. (Wuhan, China) and verified they were free of contamination through mycoplasma testing. PMSF (TIM00004‐1) and RIPA lysis buffer (strong, TIM0001) were obtained from Teusxp (Xi'an, China). Loading buffer (BL502B) and protein marker (10–180 kDa, BL712A) were obtained from Biosharp. Precast‐gel running buffer (PG00010), antibody dilution buffer (A1800), and TBST buffer (T1086) were obtained from Beijing Solarbio Science & Technology Co., Ltd. PVDF membranes (0.2 µm) for protein blotting (1620177) were obtained from Bio‐Rad Laboratories, Inc. Sponge pads for Western blotting (FFP55‐100pcs) were purchased from Beyotime, and skimmed milk powder (GC310001) was from Servicebio. Primary antibodies against NRF2 (80593‐1‐RR), KEAP1 (80744‐1‐RR), HO‐1 (81281‐1‐RR), GPX4 (82822‐2‐RR), β‐actin (81115‐1‐RR), and NF‐κB (80979‐1‐RR), and HRP‐conjugated goat anti‐rabbit IgG secondary antibody (SA00001‐2), were obtained from Proteintech Group, Inc. Chemiluminescent detection was performed using the SuperKine West Pico PLUS substrate (BMU101‐CN, Abbkine Scientific Co., Ltd.). Human TNF‐alpha ELISA Kit (GEH0004‐48T) and Human IL‐6 ELISA Kit (GEH0001‐48T) from Servicebio.

### 4.2 Characterizations

The absorbance spectra, nanozyme activity analysis of the assay, and enzyme kinetics were assessed on a multifunction microplate reader (Tecan Group Ltd., Männedorf, Switzerland). A laser scanning confocal microscope (Zeiss 980) was used for the analysis of cell and tissue slices. The morphology of N‐CDs and Ag_SA_‐CDs was observed using HR‐TEM with a JEM‐2100 instrument (JEOL, Tokyo, Japan) operating at a 200 kV acceleration voltage. Additionally, we performed Ac‐HAADF‐STEM imaging using a Thermo Scientific Spectra 300 (S)TEM instrument with picometer‐scale resolution at Hainan University. Copper grids covered with lacey carbon were used for TEM investigation, while BioTEM samples were detected using a low acceleration voltage of 80 kV. Fluorescent properties were characterized using an FP‐8650 WRE spectrofluorometer from JASCO Corporation, located in Tokyo, Japan. The levels of radicals (O_2_•^−^) were evaluated using an ESR spectrometer (Bruker EMX Plus, Germany). Thermo scientific's energy‐dispersive X‐ray fluorescence (EDXRF) system, set to 120 kV, was used for monitoring elemental identification. The XPS analysis was performed using a Thermo Fisher Scientific ESCALAB 250 spectrometer, equipped with a monochromatic Al Kα radiation source (hʋ 1486.6 eV). High‐resolution C1s, N1s, O1s, and Ag 3d core level XPS spectra were collected at 29.35 eV with a 90° take‐off angle. The flow cytometer with built‐in software (NovoCyte 2060R, Santa Clara, Agilent) was used to measure cellular ROS, uptake, and apoptosis events. All in vivo and ex vivo tests were conducted with a dual‐mode bioluminescent and fluorescent imaging system from Spectral Instruments Imaging (Kino) from Bruker USA.

### 4.3 Synthesis of Nitrogen‐Doped Carbon Dots (N‐CDs)

N‐CDs were synthesized via a bottom‐up hydrothermal molecular fusion approach by the recipe reported elsewhere with some modifications. Anhydrous citric acid (6 g, TCI, 98% purity) and urea (12 g) were dissolved in 60 mL of formic acid to form a homogeneous solution. The mixture was transferred to a polytetrafluoroethylene (PTFE)‐lined hydrothermal reactor and heated at 160°C for 4 h. Following the reaction, the resulting product was subjected to three centrifugation cycles at 13,000 rpm for 20 min each to isolate the precipitate. The collected precipitate was then freeze‐dried to yield the final N‐CD powder.

### 4.4 Synthesis of Ag_SA_‐CDs Nanozymes

To prepare Ag_SA_‐CDs, weigh 60 mg of N‐CDs and dissolve them in 24 mL of deionized H_2_O, stirring thoroughly to ensure complete dispersion. Adjust the pH of the solution to 12 using sodium hydroxide (NaOH). Add 4.8 mL of 10 mM AgNO_3_ and stir the mixture for 30 min at room temperature. Sequentially add 3.6 mL of trisodium citrate (14 mm) and 4.8 mL of NaBH_4_ (10 mM), stirring continuously to ensure homogeneity. Transfer the reaction mixture to an oil bath and incubate at 60°C for 3 h. After the reaction is complete, purify the resulting Ag_SA_‐CDs by dialysis using a 500 Da molecular weight cut‐off membrane in deionized water under light‐protected conditions to remove unreacted reagents and byproducts, freeze‐dry, and stored in a refrigerator for further use.

### 4.5 Surface Modification of T‐Ag_SA_‐CDs via T Targeting Ligand

To prepare T‐Ag_SA_‐CDs, T–COOH (0.02 mmol) was coupled to the surface of Ag_SA_‐CDs using an N‐hydroxysuccinimide (NHS) chemical method. Specifically, 1‐ethyl‐3‐(3‐dimethylaminopropyl)carbodiimide (EDC, 0.02 mmol) and NHS (0.04 mmol) were added to a solution containing TPP–COOH (0.02 mmol), and the mixture was stirred for 60 min to activate the carboxyl groups. Subsequently, 60 mg of Ag_SA_‐CDs were introduced to the activated solution, and the pH was adjusted to 7.2 to optimize the coupling reaction. The reaction mixture was stirred for 24 h at room temperature to facilitate covalent linkage. The resulting suspension was purified via dialysis against deionized H_2_O to remove unreacted reagents and byproducts, followed by freeze‐drying to yield the final T‐Ag_SA_‐CDs product.

### 4.6 Superoxide Dismutase (SOD) Activity Assay

SOD‐like activity was evaluated using the Dojindo SOD Assay Kit harmonizing to the manufacturer's instructions. Test materials were diluted in phosphate‐buffered saline (PBS) to final concentrations of 0.125, 0.25, 0.5, 1, 2, 4, and 8 µg mL^−^^1^, with three replicate wells prepared for each concentration in a 96‐well plate. Then the assay mixture was performed by incubating the samples at 37°C for 20 min, followed by immediate absorbance measurement at 450 nm using a microplate reader. The SOD inhibition rate was calculated using the formula provided in the kit manual and converted to SOD activity units (U mL^−^^1^) to quantify the enzyme activity.

### 4.7 Glutathione Peroxidase (GPx) Activity Assay

The GPx activity assay was conducted using the Beyotime Total Glutathione Peroxidase Detection Kit (Product No.: S0056) as per manufacturer's instructions, as follow. Test samples were diluted to a concentration of 10 µg mL^−^^1^ in PBS (pH 7.4), and three replicate wells were prepared for each sample. The reaction system was set up following the kit manufacturer's instructions and incubated at 37°C. Absorbance was monitored in kinetic mode using an microplate reader, with measurements taken at 340 nm at every 40 sec over a period of 14 min to assess the enzymatic activity.

### 4.8 ABTS•^+^Scavenging Activity Assay

The ABTS•^+^ stock solution was prepared 1 day in advance as follow: Mix 7 mm ABTS with 2.45 mm K_2_S_2_O₈ in a 1:1 (v/v) ratio. Allow the mixture to stand at room temperature under dark for 12 h to generate the ABTS•^+^ stock solution. Prior to use, dilute the stock solution with PBS (pH 7.4) to achieve an absorbance of 0.70 ± 0.02 at 734 nm. In a 96‐well plate, sequentially add 20 µL of sample with varying concentrations of N‐CDs and T‐Ag_SA_‐CDs (0, 2.5, 5, 7.5, 10, 12.5, 15, 17.5, 20 µg/mL) and 180 µL of ABTS•^+^ working solution to each well. Mix thoroughly, incubate in the dark for 30 min, and immediately measure the absorbance at 734 nm. Each sample was tested in triplicate, with PBS substituted for the sample as a blank control. Results are expressed as mean ± SD (*n* = 3).

The scavenging rate was calculated using the formula: The percentage inhibition was calculated using the formula:

$$\mathrm{inhibition}\left(\%\right)=\left[\left(A_{0}-A\right)/A_{0}\right]\times 100\%$$

where A_0_ represents the absorbance of the control (without Ag_SA_‐CDs) and A denotes the absorbance with Ag_SA_‐CDs or N‐CDs.

### 4.9 DPPH Radical Scavenging Assay

DPPH Radical Scavenging Assay was performed per manufacturer's instructions, as follow: First, we prepare a 0.1 mm DPPH (2,2‐diphenyl‐1‐picrylhydrazyl, Sigma–Aldrich) working solution, weigh the appropriate amount of DPPH and dissolve it in anhydrous ethanol, ensuring the solution is freshly prepared and stored in the dark to prevent degradation. In a 96‐well plate, add 20 µL of sample solutions at varying concentrations (with varying concentrations of N‐CDs and T‐Ag_SA_‐CDs (0, 2.5, 5, 7.5, 10, 12.5, 15, 17.5, 20 µg/mL prepared in PBS or with the solvent serving as the blank) to designated wells, followed by 180 µL of the DPPH working solution. Mix the contents immediately to ensure homogeneity, then incubate the plate at room temperature in the dark for 30 min. Measure the absorbance of each well at 517 nm to obtain sample readings. For controls, use 20 µL of the solvent (PBS or corresponding solvent) with 180 µL of DPPH solution as the blank control, and 20 µL of sample solution with 180 µL of anhydrous ethanol as the sample background control. Perform all measurements in triplicate wells per sample concentrations to ensure reproducibility.

The inhibition (%) was calculated using the formula:

$$\mathrm{Inhibition}\left(\%\right)=\left[1-\left(A-A_{1}\right)/A_{0}\right]\times 100\%$$

where A_0_ represents the absorbance of the blank (without N‐CDs or Ag_SA_‐CDs), A_1_ represents the absorbance of the background and A denotes the absorbance with Ag_SA_‐CDs or N‐CDs.

### 4.10 EPR Measurement of Anion (O_2_•^−^)

The background signal was produced by the immediate mixing of FeSO_4_ (1 mg mL^−^^1^) with H_2_O_2_ (5 mm) in a 1:1 volume ratio. To analyze the samples, the N‐CDs and Ag_SA_‐CDs nanozymes (1 mg mL^−^^1^) were first combined 1:1 with the background solution. Then, they were mixed 1:1 with BMPO as the spin‐trapping agent. We quickly moved the solution into the EPR device to measure it. We used a Bruker EMXplus‐6/1 spectrometer (Bruker, Germany) to record EPR spectra. The center field was 3500.00 G, the sweep width was 100.0 G, the microwave power was 6.325 mW, the power attenuation was 15.0 dB, the microwave frequency was 9.827985 GHz, the sweep time was 30.00 s, the modulation amplitude was 1.000 G, and the modulation frequency was 100.00 kHz.

### 4.11 Cell Culture

HEK293T cells (RRID: CVCL_0063) were cultured in high‐glucose DMEM supplemented with 1% streptomycin‐penicillin, and non‐essential amino acids along with 10% FBS. The cells were maintained at 37°C in a humidified incubator with 95% relative humidity and 5% CO_2_. Upon reaching approximately 90% confluence, cells were passaged using 0.25% trypsin‐EDTA to detach them from the culture surface, followed by resuspension in fresh medium for continued propagation or experimental use.

### 4.12 Cellular Uptake Assay

A density of 1 × 10^6^ cells per well was used to seed HEK293T cells in 6‐well plates, which were then incubated with 50 µg mL^−^^1^ of N‐CDs, Ag_SA_‐CDs, or T‐Ag_SA_‐CDs for 6 h. Following treatment, cells were detached using trypsin, pelleted by centrifugation at 1000 × g for 3 min, and washed twice with PBS under the same centrifugation conditions. The cells were then resuspended in 500 µL of PBS, and fluorescence histograms were acquired using a flow cytometer, with gating configured to the specific excitation and emission maxima of each carbon‐dot formulation. Data analysis was performed using the NovoCyte 2060R Flow Cytometer System and NovoSampler Pro NS200 (Agilent Technologies, Inc.) integrated software.

### 4.13 In Vitro Cytotoxicity Evaluation of T‐Ag_SA_‐CDs

The CCK‐8 assay was employed to evaluate the in vitro cytotoxicity of N‐CDs, Ag_SA_‐CDs, and T‐Ag_SA_‐CDs against HEK293T cells as follows: In a 96‐well plate, cells were placed at a density of 1×10^4^ cells/well and incubated for 24 h according to normal protocols to ensure full cell attachment. Next, a new complete medium was replaced, which included varying concentrations (ranging from 0 to 200 µg/mL) of N‐CDs, Ag_SA_‐CDs, or T‐Ag_SA_‐CDs, and incubated for another 24 h. After washed with PBS gently, each well was then filled with 100 µL fresh medium containing 10 µL CCK‐8 stock reagent, thereafter, 2 h of dark incubation at 37°C. Lastly, a microplate reader was used to detect absorbance at 450 nm. Cell viability was calculated using the following formula:

$$\mathrm{Cell}\;\mathrm{Survival}\;\mathrm{Rate}\left(\%\right)=\left(A-A0\right)/A2-A0)\times 100\%$$

where the A denoted for absorbance of the samples, A_0_ for blank that consists of cell‐free medium, and the A_2_ indicate the control group comprises cells without added nanozymes.

### 4.14 Measurement of H_2_O_2_ Scavenging Ability

The oxidative stress cell lines were prepared as follows: Cells were plated in 96‐well plates as previously outlined and treated with H_2_O_2_ (400 µm) in DMEM for 3 h. The cell culture medium was replaced with fresh complete medium containing different concentrations (0–200 µg/mL) of N‐CDs, Ag_SA_‐CDs, or T‐Ag_SA_‐CDs nanozymes, and the cells were incubated for an additional 12 h. Following by sterile PBS wash, each well were supplemented fresh medium (100 µL) with 10 µL of CCK‐8 reagent for another 2 h and measured the absorbance at 450 nm. Cell viability was determined following the established protocol.

### 4.15 In Vitro ROS Scavenging Ability

To assess the ROS scavenging capacity of N‐CDs, Ag_SA_‐CDs, and T‐Ag_SA_‐CDs in H_2_O_2_‐induced oxidative stress, HEK293T cells were seeded at a density of 5 × 10^4^ cells/well in a 12‐well plate and cultured at standard culture condition (5% CO_2_ at 37°C) for 24 h. The culture medium was then replaced with serum‐free DMEM containing 250 µM of H_2_O_2_ and incubated for 4 h to induce oxidative stress. Following this, the H_2_O_2_‐containing medium was removed, and cells were gently washed twice with PBS. Next we added fresh full DMEM with 50 µg/mL of either N‐CDs, Ag_SA_‐CDs, or T‐Ag_SA_‐CDs and let the cells sit for another 6 h. After treatment, cells were washed twice with PBS, then incubated in serum‐free DMEM containing 10 µM of DCFH‐DA dye at 37°C under dark for 30 min to detect intracellular ROS. After removing unbound DCFH‐DA by washing the cells three times with cold PBS, we immediately examined them using a confocal fluorescence microscope with Ex/Em of 488/525 nm.

### 4.16 In Vitro MitoSOX Scavenging Ability

The oxidative stress‐induced condition was produced by procedures followed the previously described ROS‐assay protocol. Briefly, after incubating cells with nanozyme nanomaterials for 6 h, the cells were gently washed twice with PBS to remove residual nanozyme. Subsequently, the cells were incubated in serum‐free DMEM supplemented with 5 µm MitoSOX Green at 37°C in the dark for 20 min to detect mitochondrial ROS. Following incubation, the cells were thoroughly rinsed three times with cold PBS to remove excess dye and immediately observed using confocal fluorescence microscopy with Ex/Em of 488/525 nm.

### 4.17 Apoptosis/necrosis Study via Annexin V‐FITC/PI Staining

To evaluate the cytotoxicity of nanozymes (N‐CDs, Ag_SA_‐CDs, and T‐Ag_SA_‐CDs) under the oxidative stress model, cells were treated with 100 µg/mL of each nanozyme for 12 h. Following incubation, the supernatant was discarded, and cells were washed twice with cold PBS to remove residual extra nanozymes. Cells were then detached using EDTA‐free trypsin, gently agitated until fully detached, and collected into flow cytometry tubes. The cell suspension was centrifuged at 400 × g for 5 min at 4°C, and the supernatant was discarded. The cell pellet was resuspended in 195 µL of 1× binding buffer, followed by the addition of 5 µL Annexin V‐FITC and 10 µL propidium iodide (PI). The mixture was gently tapped to ensure thorough mixing and incubated on ice in the dark for 20 min. Samples were immediately analyzed using a flow cytometer, with fluorescence detection at FL_1_ (530/30 nm) for Annexin V‐FITC and FL_2_ (585/42 nm) for PI to assess cell viability and apoptosis. Fluorescence histograms were recorded using FACS, and the gated events were analyzed by inbuilt software. The gate is a random set that detects the respective dyes or CDs fluorescence excitation wavelength.

### 4.18 Localization of Nanozyme in the Cytoplasm

A confocal laser scanning microscope (CLSM) evaluated the distribution of N‐CDs, Ag_SA_‐CDs, or T‐Ag_SA_‐CDs within the cellular compartment. Briefly, HEK293T cells were seeded on pre‐treated poly‐L‐lysine coverslips placed in the six‐well plate for 12 h, after treated with 50 µg mL^−^^1^ of N‐CDs, Ag_SA_‐CDs, or T‐Ag_SA_‐CDs seeded on plates and incubated at 37°C in the dark for 6 h. After two gentle washes with ice cooled PBS, replace with serum‐free DMEM containing Mito‐Tracker Green or Lyso‐Tracker Green and continue loading at 37°C in the dark for 30 min. Wash once with PBS, then fixed with 4% paraformaldehyde (w/v) at room temperature for 10 min. Lastly, the cell nuclei were dyed with DAPI (1 µg/mL) for 10 min at room temperature. The prepared cell samples were analyzed by a CLSM (Zeiss 880).

### 4.19 Cell Preparation for Transmission Electron Microscopy

Cells were cultured under standard conditions until they reached approximately 70% confluence. The culture medium was carefully removed, and any detached cells floating in the supernatant were first collected. Adherent cells were then gently trypsinized using an appropriate amount of trypsin solution. Digestion was monitored closely and terminated promptly to avoid over digestion by adding fresh complete culture medium. The cells were gently pipetted to facilitate detachment and resuspension, forming a uniform cell suspension.

The cell suspension was transferred to a sterile centrifuge tube and centrifuged at low speed (≤3000 rpm) for 3–5 min. After centrifugation, the supernatant was discarded, and the cell pellet (approximately mung bean‐sized) was retained. The cell pellet was gently resuspended in electron microscopy (EM) fixative solution, which had been pre‐equilibrated to room temperature. The cells were then fixed at room temperature in the dark for 30 min.

### 4.20 Mitochondrial Membrane Potential (ΔΨm) Assessment by JC‐1 Staining

To evaluate MMP, HEK293T cells were seeded into glass‐bottom confocal dishes and allowed to adhere for 12 h. Oxidative stress was induced, and drug treatments were applied as described for the ROS scavenging assay. Following treatment, the culture medium was replaced with fresh DMEM containing N‐CDs, Ag_SA_‐CDs, or T‐Ag_SA_‐CDs (50 µg/mL), and cells were incubated for 6 h. The cells were then gently washed with warm PBS and stained with JC‐1 probe (Beyotime, China) working solution for 20 min at 37°C in the dark, according to the manufacturer's instructions. After staining, cells were rinsed three times with PBS and immediately imaged using a CLSM (LSM 980). JC‐1 monomers were detected at 490 nm excitation and 530 nm emission, while JC‐1 aggregates were detected at 525 nm excitation and 590 nm emission. The red fluorescent dye indicated a potential‐mediated agglomeration in the mitochondrial membrane. In contrast, the green‐colored fluorescence characterized the JC‐1 monomer formation, which appeared in the depolarized mitochondrial membrane. For comparison, 10 mm CCCP (carbonyl cyanide 3‐chlorophenylhydrazone)‐treated cells were used as a positive control.

### 4.21 Intracellular ATP Measurement

Cells were seeded in 6‐well plates and exposed to oxidativestress‐induction exactly as described above. After stress, N‐CDs, Ag_SA_‐CDs, or T‐Ag_SA_‐CDs (50 µg/mL) were added and incubation was continued for 12 h. The medium was removed and 200 µL ice‐cold lysis buffer (supplied with the ATP assay kit) was added per well. After 5 min on ice, lysates were scraped, transferred to microtubes, and centrifuged at 12 000 × g, 4°C for 5 min. Supernatants were kept on ice until measurement. For the assay, 100 µL ATP detection working solution was dispensed into each well of a white 96‐well plate and equilibrated at room temperature for 3–5 min to eliminate background ATP. Subsequently, 20 µL of cell lysate was added, mixed gently with the pipette tip, and luminescence was recorded with a luminometer after a 2‐s delay. Results are expressed as relative light units (RLU).

### 4.22 Intracellular GPX Activity Assay

Cells seeded in 6‐well plates were subjected to oxidative stress induction and subsequently treated with N‐CDs, Ag_SA_‐CDs, or T‐Ag_SA_‐CDs (50 µg/mL) according to the procedures described for the ATP assay. After 12 h, remove the medium and add 200 µL of ice‐cold GPX lysis buffer to each well. Scrape cells into microcentrifuge tubes and briefly sonicate on ice. Centrifuge the lysate at 12 000×g for 10 min at 4°C. Collect the supernatant and store on ice. Immediately determine GPX activity using a commercial colorimetric assay kit (Beyotime, China) according to the manufacturer's instructions. Procedure: Mix 10 µL of lysate (containing approximately 20 µg protein) with 90 µL reaction buffer containing GSH, NADPH, and glutathione reductase. Incubate the mixture at 25°C for 10 min, then record the change in absorbance at 340 nm.

### 4.23 LDH Release Assay

The LDH Cytotoxicity Assay was performed using the LDH Cytotoxicity Assay Kit (Beyotime Biotechnology, Cat. No. C0016) according to the manufacturer's protocol to evaluate the cytotoxic effects of N‐CDs, Ag_SA_‐CDs, and T‐Ag_SA_‐CDs nanozymes on cells. To facilitate adhesion, HEK293T cells were planted in 96‐well plates at an optimum density (5 × 10^3^ cells/well) and incubated overnight. The cells were then treated with 50 µg/mL of N‐CDs, Ag_SA_‐CDs, and T‐Ag_SA_‐CDs or 200 µM H_2_O_2_, for 24 in complete culture medium. Untreated cells served as the low control (spontaneous LDH release), while cells treated with 0.1% Triton X‐100 served as the high control (maximum LDH release). Culture supernatants (100 µL/well) were collected and transferred to a new 96‐well plate. LDH reaction solution (100 µL/well) from the kit was added to each well, and the plate was incubated at room temperature for 30 min in the dark. The reaction was stopped by adding the stop solution (10 µL/well), and absorbance was measured at 490 nm using a microplate reader. Background absorbance from the culture medium alone was subtracted from all samples. Cytotoxicity was calculated using the following formula:

$$\mathrm{Cytotoxicity}=\left[A-A_{0}\right)/A_{1}-B_{0}]$$

where A_0_ represents the absorbance of the blank (without Ag_SA_‐CDs), A_1_ represents the absorbance of the background, A denotes the absorbance with Ag_SA_‐CDs or N‐CDs and B_0_ denoted as low control.

### 4.24 Western Blot Analysis

Cells underwent treatment with N‐CDs, Ag_SA_‐CDs, or T‐Ag_SA_‐CDs, followed by the collection and clarification of culture supernatants through centrifugation. Protein samples (6× concentrated) were separated using Epizyme precast gels and subsequently transferred to methanol‐activated PVDF membranes (Immun‐Blot, Bio‐Rad) at 4°C. Membranes were incubated with 5% non fat milk at room temperature for 2 h to block, followed by overnight incubation at 4°C with primary antibodies targeting KEAP1, NRF2, HO‐1, GPX4, NF‐κB, or β‐actin. Following washing (4 × 10 min with TBST), membranes were incubated with HRP‐conjugated secondary antibody for 1 h at room temperature. Protein bands were visualized with the ChampChemi 910 Plus imaging system (SinSage Technology Co. Ltd.) and quantified in relation to β‐actin.

### 4.25 Measurement of Cytokine Secretion by ELISA

After treatment with N‐CDs, Ag_SA_‐CDs, or T‐Ag_SA_‐CDs, the culture supernatants were collected and clarified by centrifugation to remove cell debris for downstream analyses. Following treatment, the cells were treated with 250 µMM H_2_O_2_ for 4 h. Then, the culture supernatants were collected, and the concentrations of excreted proinflammatory cytokines (TNF‐α, IL‐6) were quantified via the corresponding commercial ELISA Kit (ServiceBio, China) according to the manufacturer's instructions.

#### 4.25.1 Cisplatin‐Induced Acute Kidney Injury (AKI) Mouse Model

Male C57BL/6 mice (8–10 weeks) received a single tail‐vein injection of cisplatin (CDDP, 20 mg kg^−^^1^, dissolved in 0.9% saline). Mice were sacrificed at 24, 48 and 72 h post‐injection; kidney tissues and serum were collected for subsequent histological and serum biochemical analyses to confirm successful establishment of AKI.

#### 4.25.2 Biodistribution Study

AKI mice were randomly divided into three groups and intravenously injected with N‐CDs, Ag_SA_‐CDs, or T‐Ag_SA_‐CDs at 10 mg kg^−^^1^. Animals were euthanized at 1, 3, 6, 12, and 24 h post‐injection; brain, heart, spleen, liver, lung, and kidney were harvested. Fluorescence images of whole organs were acquired using a Kino small‐animal imager (Spectral Instruments Imaging, USA) with excitation/emission filters set according to the emission maxima of each carbon dot.

### 4.26 Pharmacokinetic Study of N‐CDs, Ag_SA_‐CDs, and T‐Ag_SA_‐CDs

For pharmacokinetic analysis, N‐CDs, Ag_SA_‐CDs, and T‐Ag_SA_‐CDs (200 µL, concentration 1 mg/mL) were dissolved in physiological saline and administered to mice (*n* = 3) via intravenous injection. Blood samples (∼50 µL) were collected from the orbital sinus at 0.5, 1, 1.5, 2, 2.5, 3, 6, 12, and 24h post‐injection. Each blood sample was mixed with 100 µL lysis buffer containing 1% Triton X‐100 (Sigma, USA), followed by incubation overnight with 0.5 mL DMSO. After centrifugation at 15 000 rpm for 10 min, the fluorescence intensity of the supernatant was measured using a microplate reader (Ex/Em = 560/650 nm). The obtained fluorescence values were used to quantify the concentrations of N‐CDs, Ag_SA_‐CDs, and T‐Ag_SA_‐CDs in blood, and to construct concentration–time profiles for pharmacokinetic analysis.

### 4.27 Hemolysis Assay

Whole blood was collected from healthy BALB/c mice via tail vein, orbital sinus, or cardiac puncture and anticoagulated with EDTA. RBCs were isolated by centrifugation (3 000 rpm, 15 min), washed three times with PBS, and resuspended at ∼5% v/v. For the assay, 20 µL RBC suspension was mixed with 1 mL of test nanomaterial solution (25, 50, 100, 200, 400, 800, 1000 µg/mL T‐Ag_SA_‐CDs in PBS, pH 7.4), PBS (negative control), or ultrapure water (positive control) in triplicate. Samples were incubated at 37°C for 4 h, centrifuged (3 000 rpm, 15 min), and 200 µL of supernatant was transferred to a 96‐well plate to measure absorbance at 542 nm. The hemolysis (%) rate was calculated as $\left(OD_{sample}-OD_{PBS}\right)/\left(OD_{dGH_{2}O}-OD_{PBS}\right)\times 100\%$.

#### 4.27.1 Quantitative Biodistribution of T‐Ag_SA_‐CDs in AKI Mice

AKI mice received a single intravenous dose of T‐Ag_SA_‐CDs (10 mg kg^−^^1^) and were euthanized at 1, 3, 6, 12, or 24 h post‐injection (*n* = 3 per time point). Heart, brain, liver, spleen, lung and kidney were excised, weighed, and homogenized in PBS (1:3 w/v). Homogenates were centrifuged (12 000 × g, 10 min, 4°C) and the fluorescence intensity of the supernatant was measured (Ex 560 nm / Em 640 nm). Concentrations were calculated from a standard curve prepared in control tissue homogenate. Biodistribution is expressed as % injected dose per gram tissue (% ID g^−^^1^) = [(fluorescence‐derived CD amount per gram tissue) / (total injected dose)] × 100%.

#### 4.27.2 In Vivo Therapeutic Evaluation of Nanozyme Against Cisplatin‐Induced AKI Models

Male C57BL/6 mice were randomly distributed into 6 groups (*n* = 6): (1) healthy PBS; (2) AKI + PBS; (3) AKI + N‐acetylcysteine (NAC); (4) AKI + N‐CDs; (5) AKI + Ag_SA_‐CDs; (6) AKI + T‐Ag_SA_‐CDs. AKI was induced with a single tail‐vein injection of cisplatin (20 mg kg^−^^1^). Treatments were administered intravenously 1 h before cisplatin and then repeated at 24 h and 48 h (total three doses for 10 mg kg^−^^1^; NAC dose equivalent to T‐Ag_SA_‐CDs). Mice were euthanized at 72 h; blood was collected, centrifuged (4°C, 3 000 × g, 20 min), and serum sent to the Wuhan Servicebio Technology Co., Ltd for further automated measurement of BUN and creatinine. Kidneys tissues slices were fixed in 4% paraformaldehyde, paraffin‐embedded, sectioned (4 µm) and stained with H&E for histopathological evaluation under light microscopy.

Paraffin‐embedded kidney sections (4 µm) were deparaffinized, rehydrated, and permeabilized with 20 µg mL^−^^1^ of Proteinase K for 15 min at 37°C. After PBS rinses, apoptotic cells were labeled with TUNEL reaction mixture (Beyotime, China) for 60 min at 37°C in the dark. Sections were washed three times with PBS, counterstained with DAPI, and imaged under a fluorescence microscope.

Paraffin sections (4 µm) were dewaxed in eco‐clear (3 × 10 min), rehydrated through graded ethanol to water, and subjected to microwave antigen retrieval in EDTA buffer. After cooling and three PBS washes, tissues were circled with a hydrophobic pen, blocked with 5% BSA (30 min, RT), and incubated overnight at 4°C with primary antibody. Following three PBS washes, Alexa‐Fluor‐conjugated secondary antibody was applied (50 min, RT, dark). Sections were counterstained with DAPI (10 min), treated with autofluorescence quencher (5 min), rinsed, mounted with anti‐fade medium, and imaged using DAPI and FITC channels.

#### 4.27.3 Statistical Methods

Flow cytometric analyses were performed with NovoCyte 2060R, Agilent software. All statistical analyses, including one‐way ANOVAs were conducted using OriginPro 2022 software. ^*^ *p *< 0.05, ^**^ *p *< 0.01, ^***^ *p *< 0.001, ^****^ *p *< 0.0001 showing the corresponding significant levels. All error bars represent the mean ± SEM unless stated otherwise. Bodyweight and quantitaitive biodistribution analysis were conducted using Microsoft Excel 2016.

## Conflicts of Interest

The authors declare no conflict of interest.

## Supporting information




**Supporting File**: advs73717‐sup‐0001‐SuppMat.pdf.

## Data Availability

The data that support the findings of this study are available in the supplementary material of this article.
