# Supplementary material for: Precision‐Engineered Silver Single‐Atom Carbon Dot Nanozymes for Theranostic Management of Acute Kidney Injury
Source: Adv Sci (Weinh). 2026 Jan 15;13(16):e19393. doi: 10.1002/advs.202519393 (PMC13042797; doi:10.1002/advs.202519393)
Supplement: Supplementary file 1 — Supporting File: advs73717‐sup‐0001‐SuppMat.pdf. [file ADVS-13-e19393-s001.pdf]

Supporting Information

Precision-Engineered Silver Single-Atom Carbon Dot Nanozymes for Theranostic Management of Acute Kidney Injury

Tianle Tang†, Jun Zhang†, Yan Wang, Guoping Chen, Kehui Yuan, Yuan He, Chenchen Li. Sumaira Hanif, Yang Yang, Yanli Wang and Pir Muhammad**

Prof. T. Tang, J. Zhang, Y.He, Dr. S. Hanif

NHC Key Laboratory of Tropical Disease Control, School of Life Sciences and Medical Technology, Hainan Medical University, Haikou, Hainan, 571199, China.

Y. Wang, Dr. C. C. Li, Dr. Y. Yang, Prof. Y. Wang, Prof. P. Muhammad,

Engineering Research Center of Tropical Medicine Innovation and Transformation of Ministry of Education, International Joint Research Center of Human-machine Intelligent Collaborative for Tumor Precision Diagnosis and Treatment of Hainan Province, School of Pharmacy, Hainan Academy of Medical Sciences, Hainan Medical University, Haikou, Hainan 571199, China.

E-mail: wangyanli@muhn.edu.cn (YL. Wang)

E-mail: pir@muhn.edu.cn (P. Muhammad)

G. Chen, K. Yuan

The First Affiliated Hospital of Hainan Medical University, Hainan Medical University, Haikou 571199, China.

***Corresponding Authors:**

E-**mail:** wangyanli@muhn.edu.cn (YL. Wang); pir@muhn.edu.cn (P. Muhammad)

Methods

In Situ DRIFT Assay of enzyme mimics activities

In situ enhanced DRIFT spectra were collected through a Nicolet 6700 FT-IR spectrometer (Nicolet USA), with a wavenumber range of 400-4000 cm^{-1}, a signal-to-noise ratio of 50000:1. Briefly, stock solution of catalyst (solution A) was prepared by dispersing N-CDs or Ag_{SA}-CDs (1 mg mL^{-1} in PBS, pH 7.4) were employed as the catalytic materials for the SOD-like activity assay. In the meantime, Solution B consisted of 0.4 mM xanthine in PBS (pH 7.4), and Solution C was prepared by diluting xanthine oxidase to 0.1 U mL^{-1} in the same buffer. For each measurement, 15 μL of Solution A, 135 μL of Solution B, and 10 μL of Solution C were combined to initiate the reaction. In situ infrared spectra were collected at room temperature over a period of 0 to 20 minutes to monitor the catalytic conversion and assess the SOD-like behavior of the samples.

For the in situ infrared measurements of estimation of GPX-like Activity, three stock solutions were prepared in PBS buffer (pH 7.4): Solution A containing N-CDs or Ag_{SA}-CDs (1 mg/mL), Solution B containing GSH at 100 mM, and Solution C containing H_2O_2 (20 mM). For each assay, 125 μL of PBS (pH 7.4) was combined with 15 μL of Solution A, followed by 10 μL of Solution B and 10 μL of Solution C to initiate the reaction. The mixture was immediately transferred to the DRIFTS cell, and spectra were collected from 0 to 20 minutes at room temperature to monitor time-dependent changes associated with the GPx-like catalytic process.

Determination of Ag^+ release

The release of silver ions (Ag^+) was measured using the TMB (3,3',5,5'-tetramethylbenzidine) colorimetric method. $AgNO_3$ standard solutions (0, 0.05, 0.1, 5,

10, 50, and 100 μg/mL) were prepared in a pH 4.5 sodium acetate buffer. For each standard, 800 μL was mixed with 200 μL of 1 mM TMB, incubated at room temperature in the dark for 15 min, and the absorbance at 655 nm was measured to construct the Ag^+ standard curve. Ag_{SA}-CDs solution (1 mg/mL) was incubated in PBS (pH 4.5, 6.5, 7.5), DMEM, and DMEM supplemented with 10% FBS. At 1, 24, 48, and 72 h, 100 μL samples were withdrawn, mixed with 700 μL pH 4.5 sodium acetate buffer, and then 200 μL of 1 mM TMB was added. After 15 min incubation at room temperature, the absorbance at 655 nm was measured, and the Ag^+ concentration was determined using the standard curve.

Immunohistochemistry (IHC) and TUNEL assay

The predetermined dissected Tumor or organ samples were fixed with 10% neutral buffered formalin, subsequently paraffinized for histological examination. All 4 mm sections of the tissue samples were stained with H&E to observe the histological changes. Damaged AKI tissues were also counterstained by using immunofluorescence. IHC for TUNEL assay, GPX4, HO-1, and NRF2 were conducted serially for apoptotic cell death in AKI tissues as instructed by the manufacturer's manual.

The EXAFS signals fitting and wavelet transform (WT) analysis

We used Athena software (version 0.9.26) to calibrate the post-edge and pre-edge lines and perform background corrections on the XAFS data.[1] Next, we employed Artemis (version 0.9.26) to conduct Fourier-transformed fitting. [2] For the k^3-weighted fitting, we selected a k-range of 3 to 12 $Å^{-1}$ and an R-range of 1 to 3 Å. We modeled the scattering paths using reference standards, including Ag foil, $AgNO_3$, Ag_2S, and Ag_2O. The fitting process involved four key parameters: energy shift (E_0), coordination number (CN), bond length (R), Debye-Waller factor (σ^2), and energy offset (ΔE_0). The

χ(k) data exported from Athena was then analyzed using the Hama Fortran code for Wavelet Transform analysis. To capture the overall distribution, we applied the following parameters: an R-range of 0 to 6 Å, a k-range of 3 to 13.9 $Å^{-1}$, a k^3 weight, and a Morlet function (κ=10, σ=1) as the mother wavelet.[3]

DFT calculation method

All density functional theory (DFT) calculations[4-5] were carried out in the CP2K code.[6] All calculations employed a mixed Gaussian and planewave basis sets. Core electrons were represented with norm-conserving Goedecker-Teter-Hutter pseudopotentials,[7-9], and the valence electron wavefunction was expanded in a double-zeta basis set with polarization functions[10] along with an auxiliary plane wave basis set with an energy cutoff of 450 Ry. The generalized gradient approximation exchange-correlation functional of Perdew, Burke, and Enzerhof (PBE)[11] was used. Each configuration was optimized with the Broyden-Fletcher-Goldfarb-Shanno (BGFS) algorithm with SCF convergence criteria of 1.0×10^{-5} au. The van der Waals correction of Grimme's DFT-D3 model was also adopted.[12]

In the present work, Gibbs free energy change (ΔG) for each elementary step was calculated as below:[13-14]

$$\Delta G = \Delta E_{elec} + \Delta E_{ZPE} - T\Delta S \quad (1)$$

where E_{elec} and E_{ZPE} are the electronic term, which is directly derived from DFT calculation, and zero-point energy (ZPE) contribution, respectively. S is the entropy and T is the temperature (298.15 K). E_{ZPE} and TS were calculated using the following equations for each reaction intermediates[15],

$$E_{ZPE} = \frac{1}{2} \sum_i h\nu_i \quad (2)$$

$$-TS = k_B T \sum_i \ln\left(1 - e^{-\frac{hv_i}{k_B T}}\right) - \sum_i hv_i \left(\frac{1}{e^{\frac{hv_i}{k_B T}} - 1}\right) \qquad (3)$$

where k_B is the Boltzmann constant, h is Planck's constant and v_i is vibrational frequencies. The localized harmonic oscillator approximation with a displacement of 0.01 Å was used in the vibrational frequency calculations. During vibrational frequency calculation, only the reaction intermediates were relaxed while all other atoms of the framework were fixed.

Supporting Figures

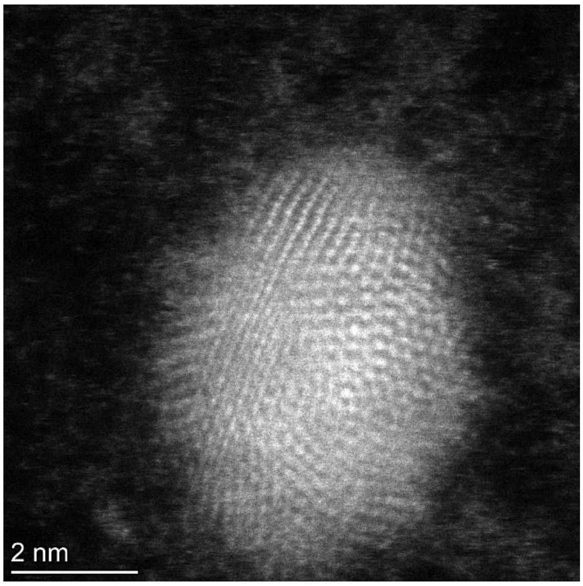

Figure S1. Ac-HAADF-STEM image of Ag nanocluster with a single atom of Ag dispersed on an amorphous N-CDs structure.

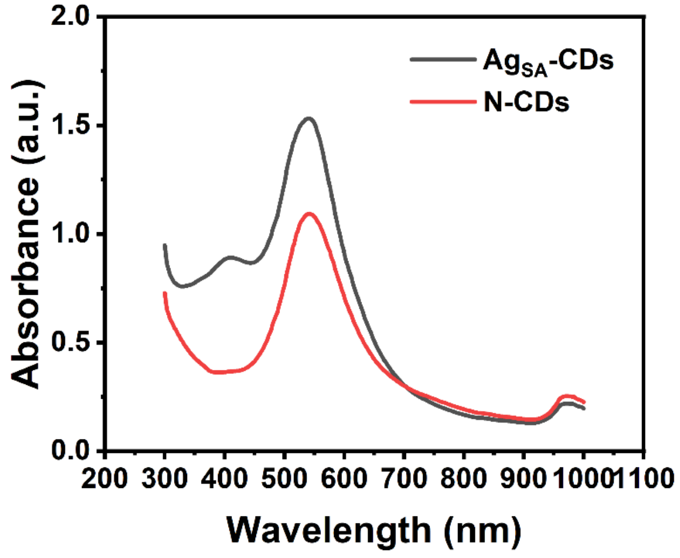

Figure S2. UV-visible spectra of N-CDs and Ag$_{SA}$-CDs in H_2O.

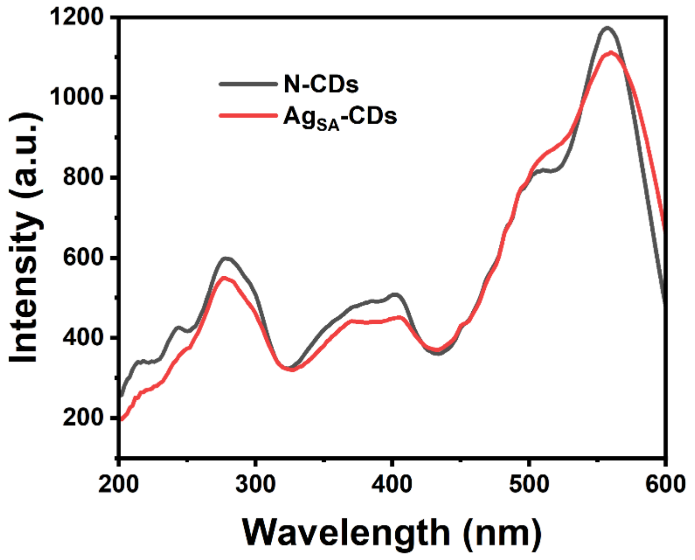

Figure S3. Full-scale FL intensity of the N-CDs and Ag$_{SA}$-CDs, showing the stability of the signal intensity and variation after Ag dopant.

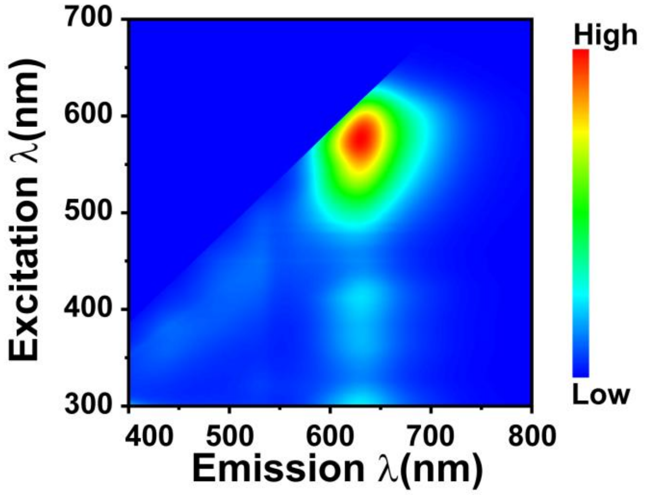

Figure S4. Excitation-emission 3D matrices of the N-CDs nanozyme.

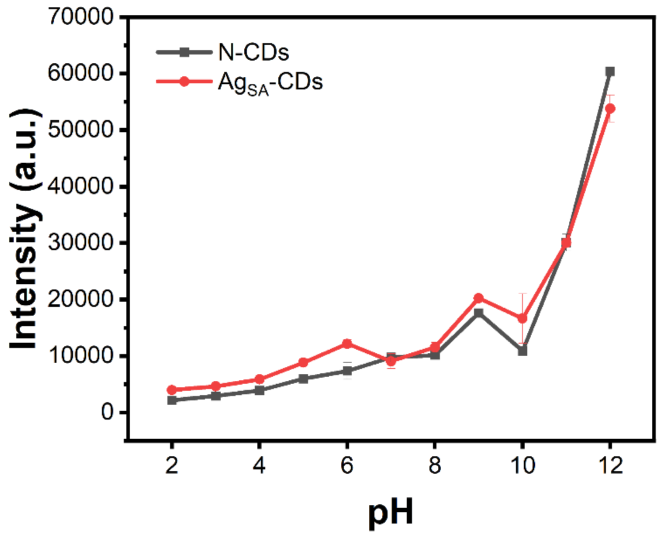

Figure S5. The effect of pH on fluorescence intensity variations (n=3).

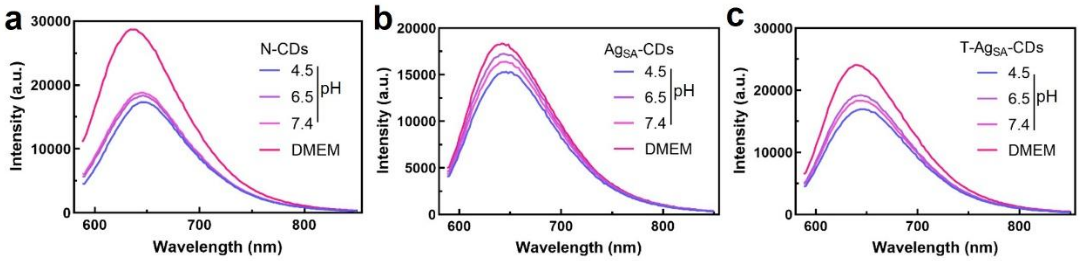

Figure S6. Stability of fluorescence emission of the nanozymes in pH- and cell culture medium-independent way. Steady-state emission spectra (λex = 560 nm) of (a) N-CDs, (b) Ag_{SA}-CDs, and (c) T-Ag_{SA}-CDs measured in buffers at pH 4.5, 6.5, 7.4, and complete DMEM cell culture medium (pH ≈ 7.4).

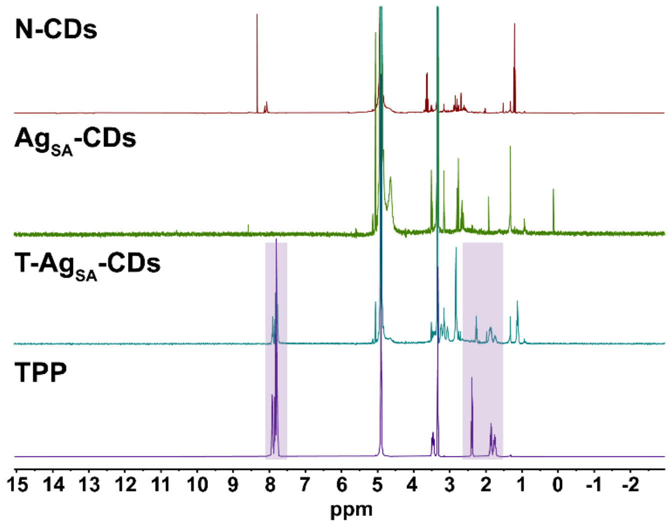

Figure S7. NMR spectra of N-CDs, Ag_{SA}-CDs, T-Ag_{SA}-CDs, and TPP in d-CH_3OH.

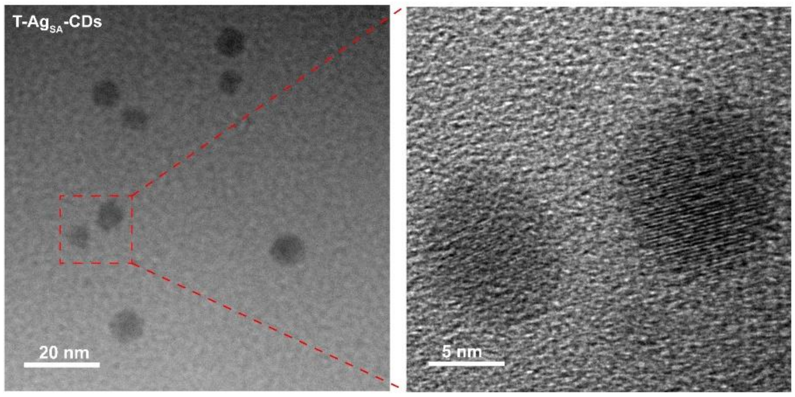

Figure S8. Representative TEM image of T-Ag_{SA}-CDs is shown, with an enlarged section displayed in high-resolution TEM mode.

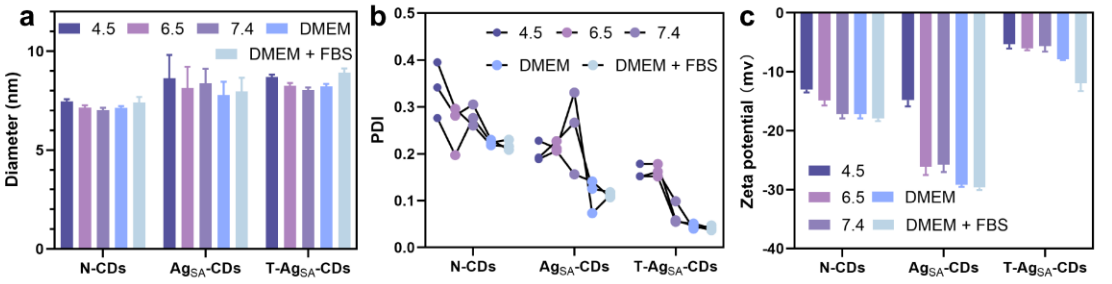

Figure S9. The DLS data (a) PDI, polydispersity index (b), and zeta potential (c) showing the stability of N-CDs, Ag_{SA}-CDs, and T-Ag_{SA}-CDs in FBS and DMEM+10% FBS. Data were presented in 3 replicate runs (n=3).

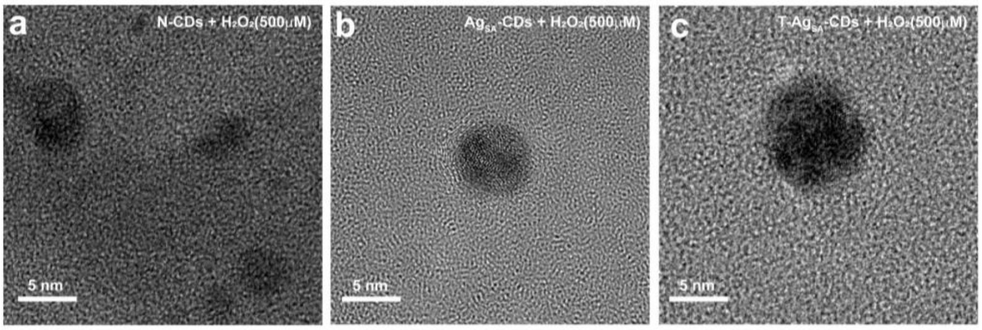

Figure S10. TEM images of N-CDs, Ag_{SA}-CDs, and T-Ag_{SA}-CDs under H_2O_2-stimulated ROS environmental conditions.

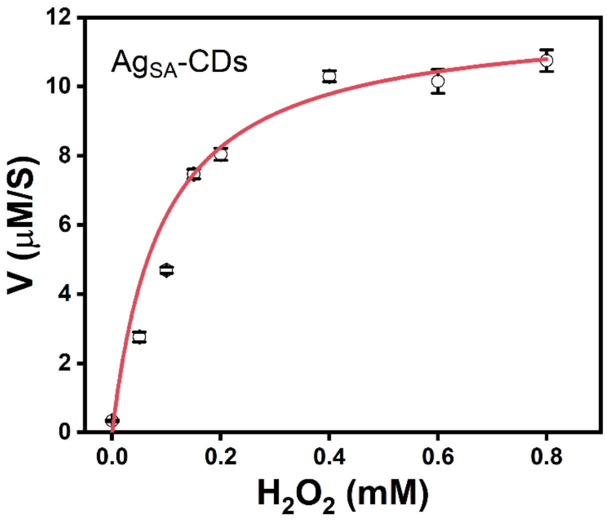

Figure S11. GPx-like activity of Ag_{SA}-CDs nanozymes. The red line represents the Michaelis-Menten curve fitting for GPx-like activity of Ag_{SA}-CDs nanozyme. (Bars represented means ± SD, n = 3 each).

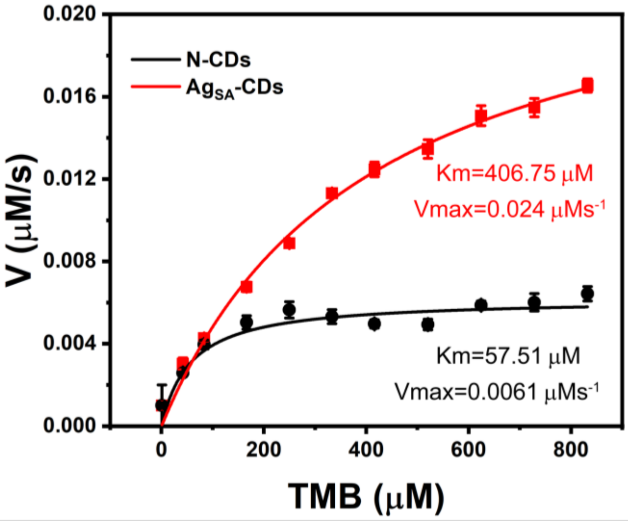

Figure S12. Kinetics for POD-like at varying concentrations of TMB substrates. The line represents the Michaelis-Menten curve fitting for POD-like activity of Ag_{SA}-CDs and N-CDs nanozyme. Error bars represent standard deviations (n = 3).

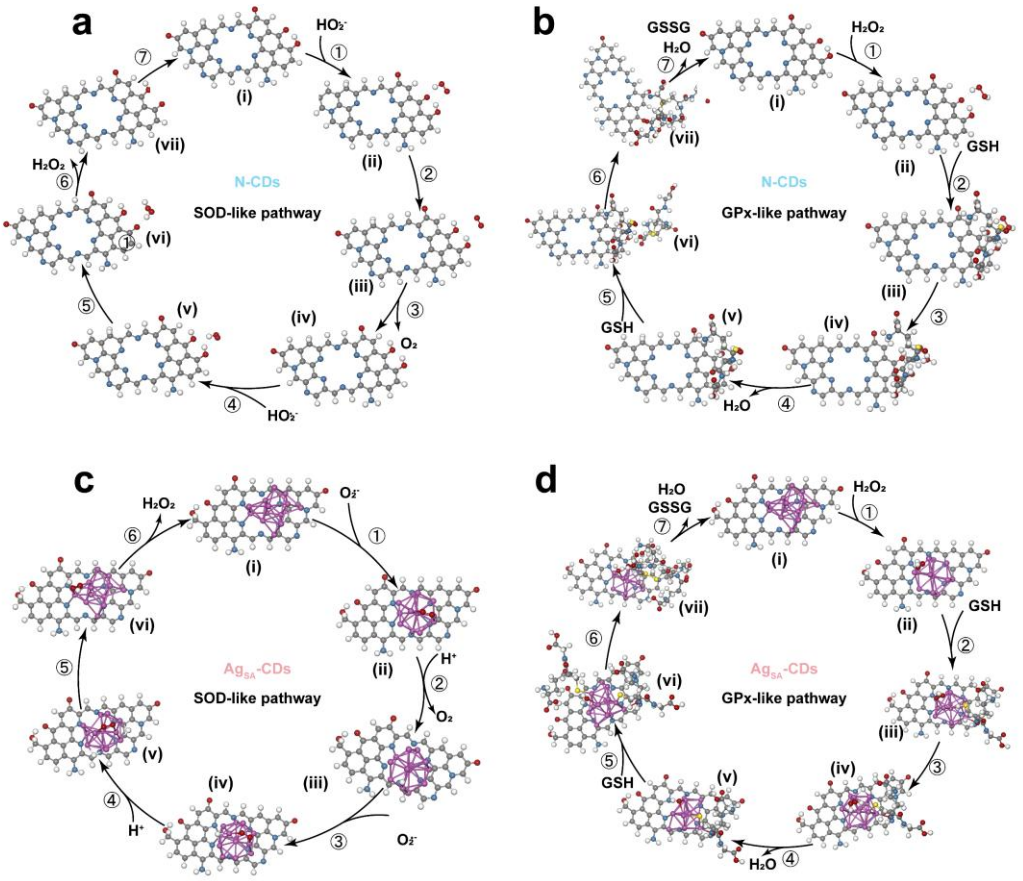

Figure S13. (a-d) Proposed reaction pathways on N-CDs and Ag_{SA}-CDs models for SOD-like and GPx-like pathways.

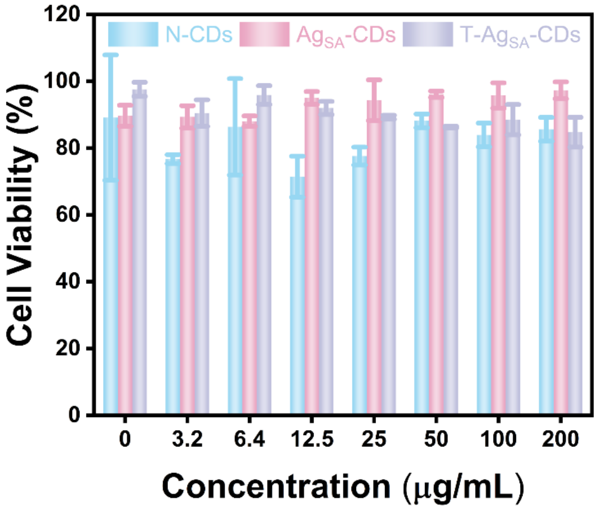

Figure S14. Effect of N-CDs, Ag_{SA}-CDs, and T-Ag_{SA}-CDs on cell viability against 293T cells without oxidative stress conditions. (Bars represented means ± SD, n = 3 each).

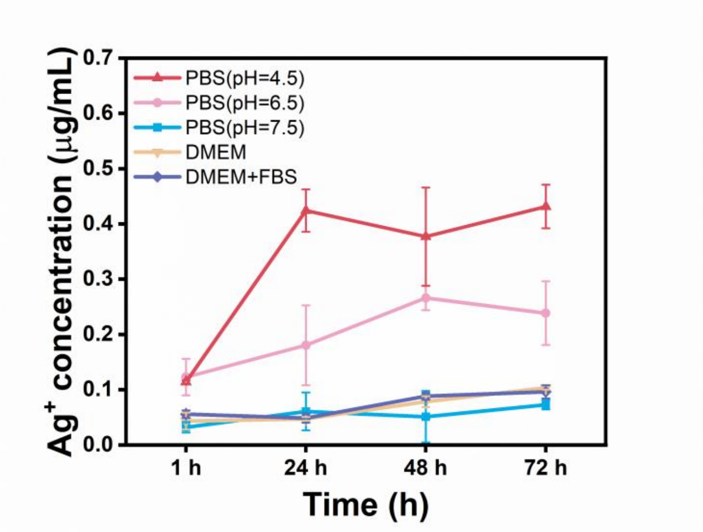

Figure S15. Ag^{+} release profiles of Ag_{SA}-CDs in PBS (pH 4.5, 6.5, and 7.5), DMEM, and DMEM containing 10% FBS. (Error bars represented means ± SD, n = 3 each).

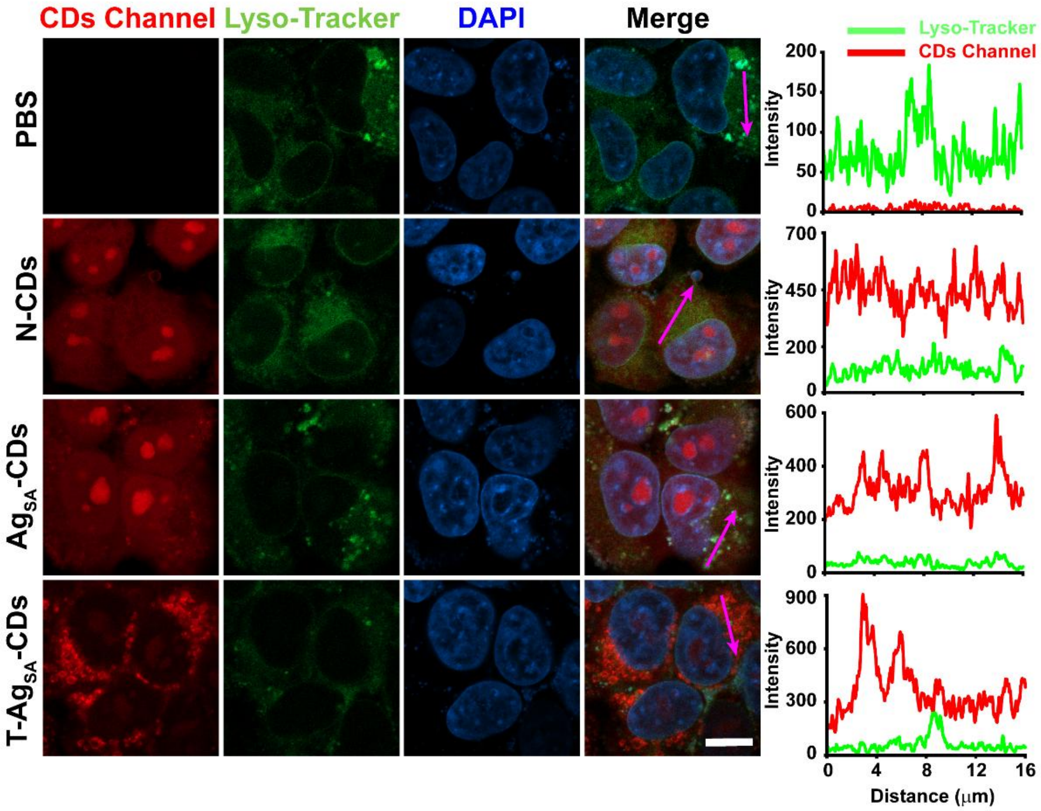

Figure S16. Confocal images of the co-localization between N-CDs, Ag$_{SA}$-CDs, and T-Ag$_{SA}$-CDs and lysosome in HEK293T cells (lysosome stained by lysotracker (green), Nuclei stained by DAPI (blue), red-emissive CDs (red), (Scale bar: 10 μm).

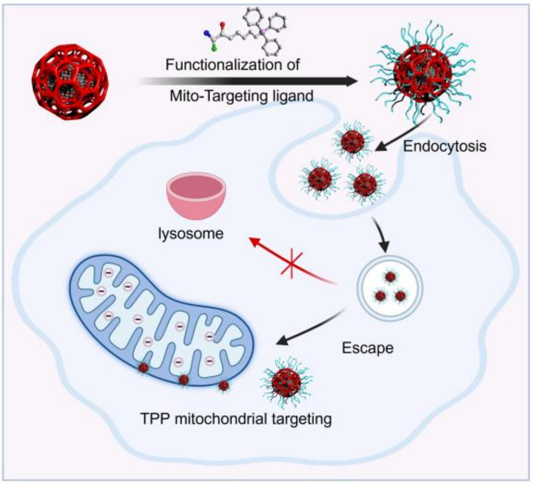

Scheme S1. Mechanism of cellular internalization, lysosomal escape, and mitochondrial targeting of T-Ag$_{SA}$-CDs (TPP-functionalized Ag$_{SA}$-CDs).

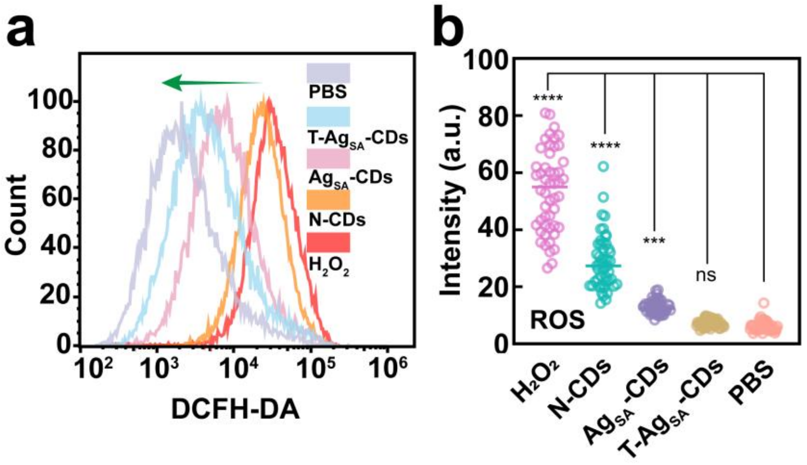

Figure S17. a) Flow cytometric histogram of DCFH-DA (Excitation/Emission, 488/520 nm) in HEK293T after treatment with nanozymes (50 μg/mL). The green arrow indicated the shifting of ROS from high to low intensity. b) Quantitative data were derived from the CLSM images of ROS depicted in Figure 5d, utilizing ImageJ software across 3 replicate experimental data (n=3).

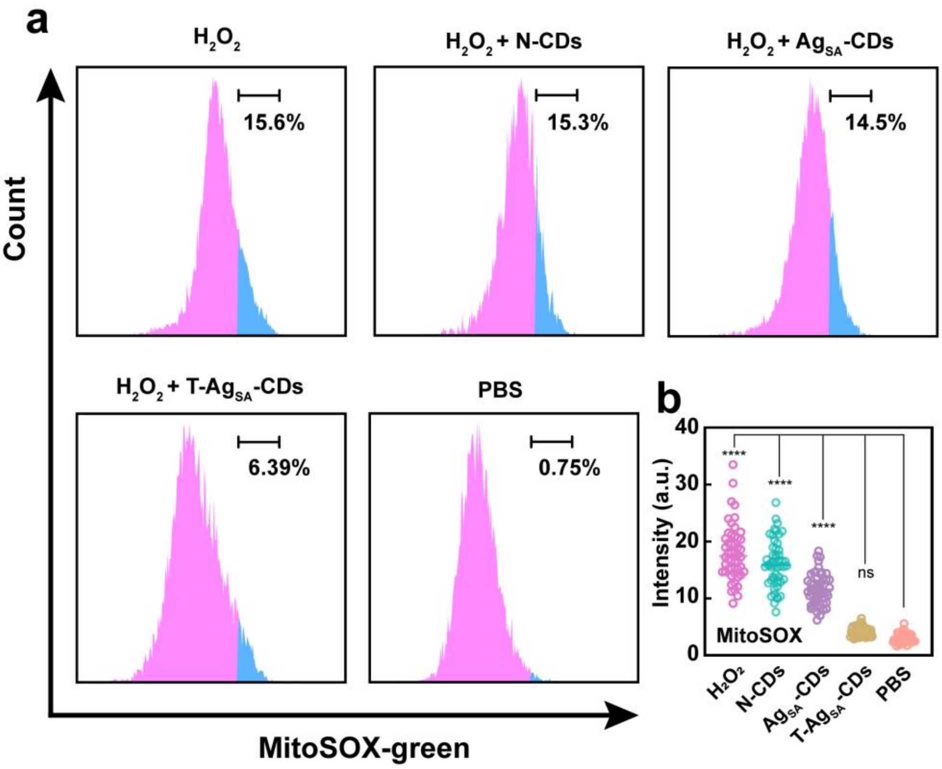

Figure S18. a) Flow cytometric histogram of MitoSOX green (Excitation/Emission, 488/510 nm) in HEK293T after treatment with nanozymes (50 μg/mL). b) Quantitative data were derived from the CLSM images of MitoSOX presented in Figure 5e using ImageJ software across 3 replicate experimental analyses (n=3).

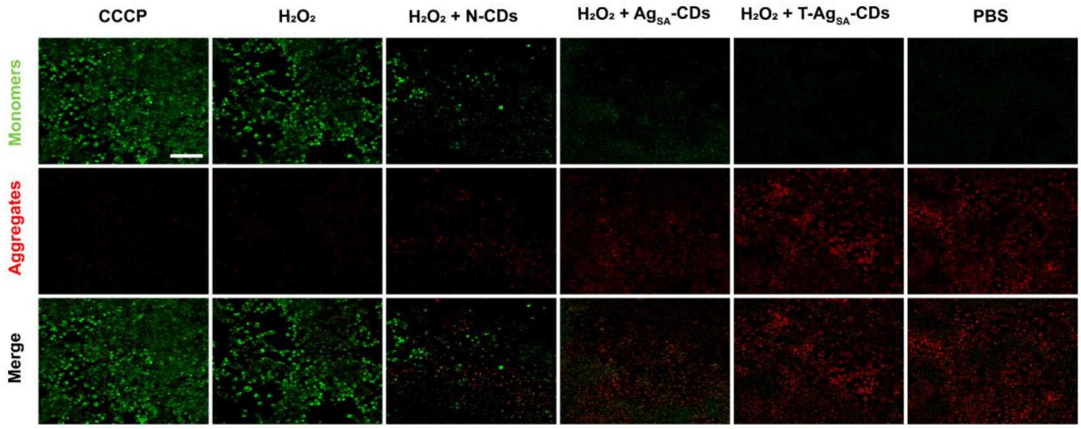

Figure S19. JC-1 analysis of HEK293T cells as a measure of mitochondrial transmembrane potential ($\Delta\Psi m$) changes in various treatments (H_2O_2, H_2O_2+N-CDs, H_2O_2+Ag$_{SA}$-CDs, and H_2O_2+T-Ag$_{SA}$-CDs-treated cells. The color shows JC-1 aggregates (red) in mitochondria and JC-1 monomers (green) in the cytosol, scale bar 100 μm.

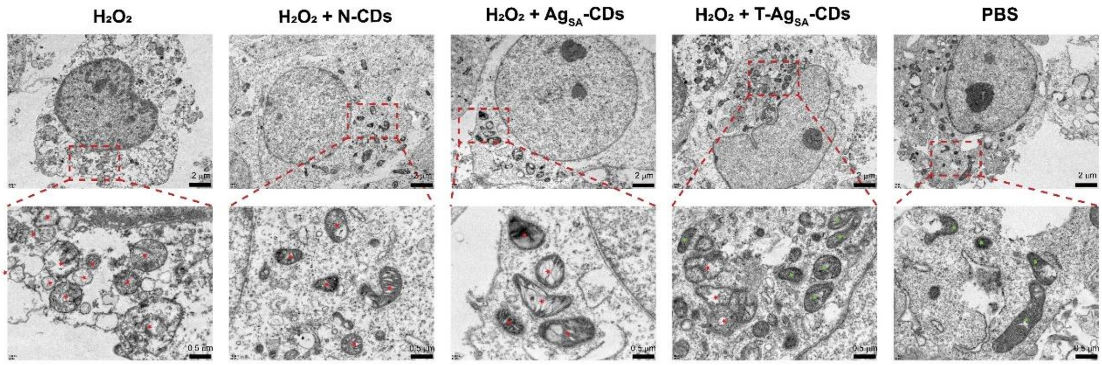

Figure S20. Bio-TEM images of HEK293T cells under different treatments: H_2O_2 alone, H_2O_2 with N-CDs, H_2O_2 with Ag$_{SA}$-CDs, H_2O_2 with T-Ag$_{SA}$-CDs, and untreated control. Scale bar: 2 μm. Enlarged regions are shown in the lower panels with a scale bar of 0.5 μm.

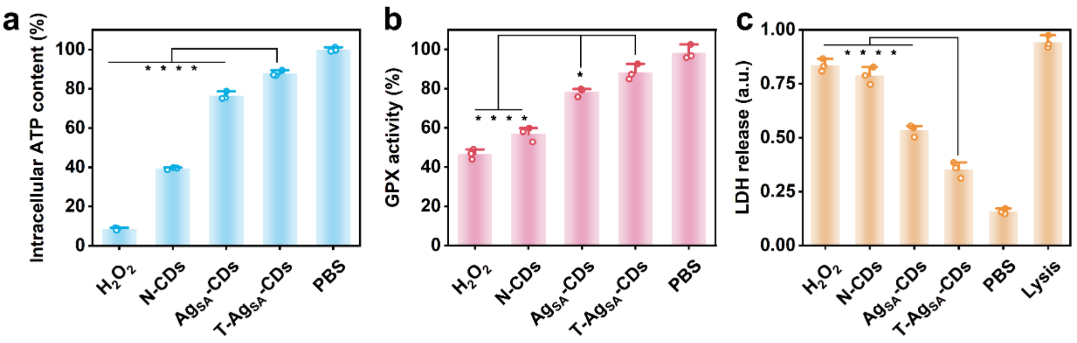

Figure S21. Expression of intracellular ATP (a), GPX activity (b), and LDH release (c) upon treatment of CDs nanozyme under H_2O_2 to mitigate oxidative stress. The data presented were a replicate of 3 consecutive runs (n=3).

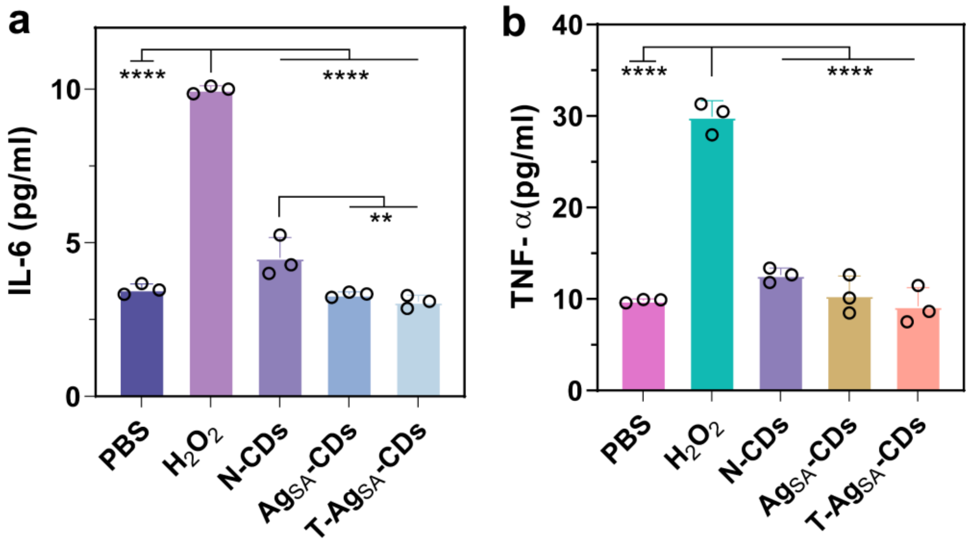

Figure S22. IL-6 (a) and TNF-α (b) expression level after treatment of HEK293T cells with and without H_2O_2, a stimulated oxidative stress environment.

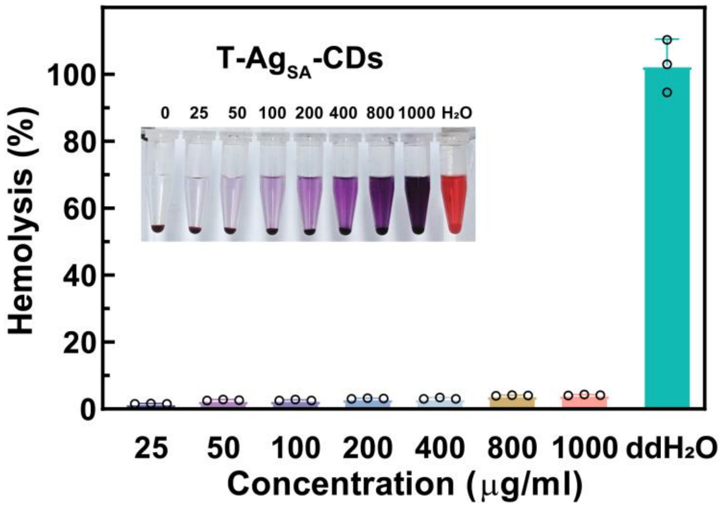

Figure S23. Hemolysis evaluation of T-Ag$_{SA}$-CDs nanozyme at different concentrations (0–1000 μg mL^{-1}). Inset: photographs of RBC suspensions showing no visible hemolysis across concentrations, while ddH$_2$O served as the positive control.

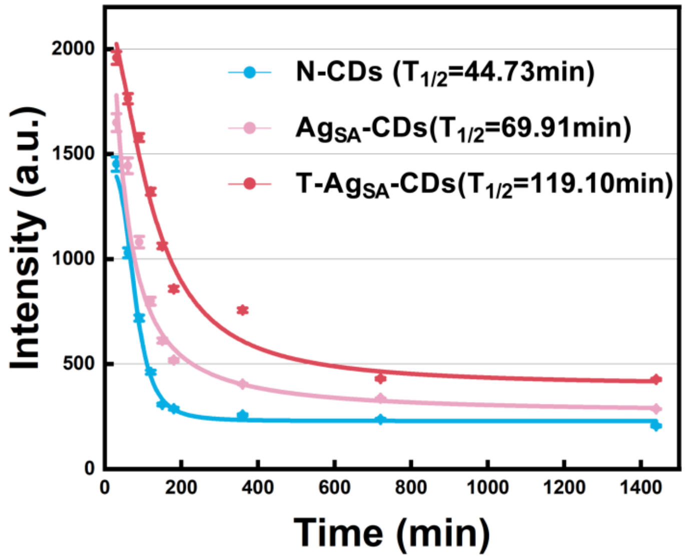

Figure S24. The blood circulation curve obtained from fluorescence intensities (subtracted background signal) of CDs measured after i.v. injected N-CDs, Ag$_{SA}$-CDs and T-Ag$_{SA}$-CDs (n = 3) and fluorescence intensity were recorded at Ex/Em = 560/650 nm. The distribution half-life of N-CDs, Ag$_{SA}$-CDs, and T-Ag$_{SA}$-CDs was calculated by exponential decay fitting-1 and was 44.73, 69.91, and 119.10 minutes, respectively.

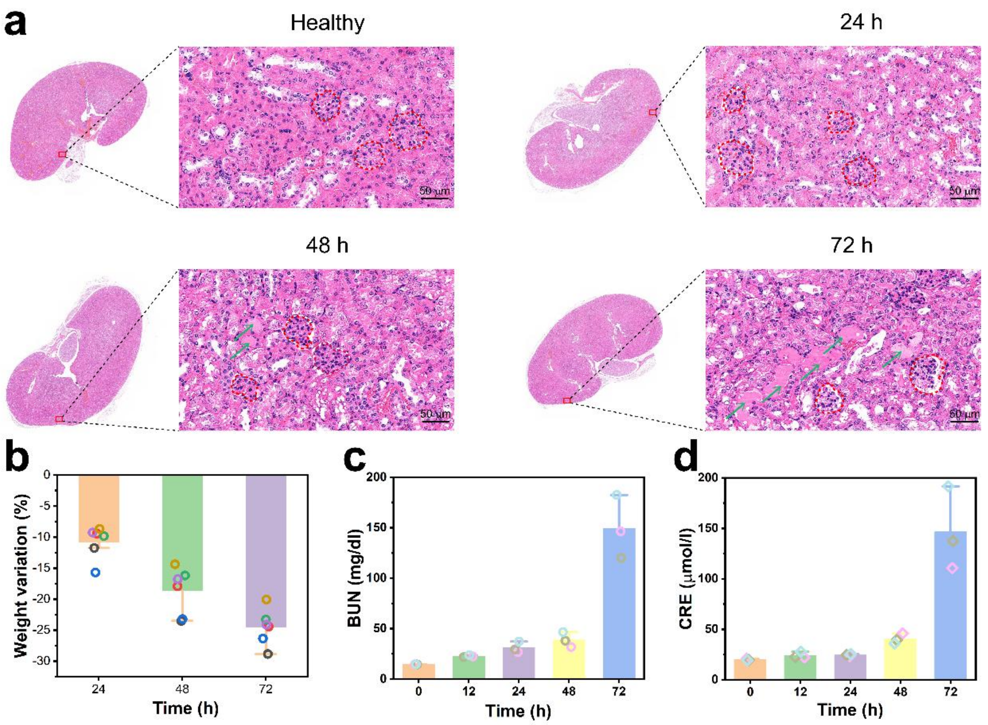

Figure S25. Development of successful cisplatin-induced AKI model. (a) Representative H&E-stained kidney sections from AKI mice at 24, 48, and 72 h post-cisplatin injection (scale bar: 50 μm). (b) Body weight changes, (c) blood urea nitrogen (BUN), and (d) serum creatinine (CRE) levels of control and AKI mice at the indicated time points. Data are presented as mean ± SD (n = 3 independent animals).

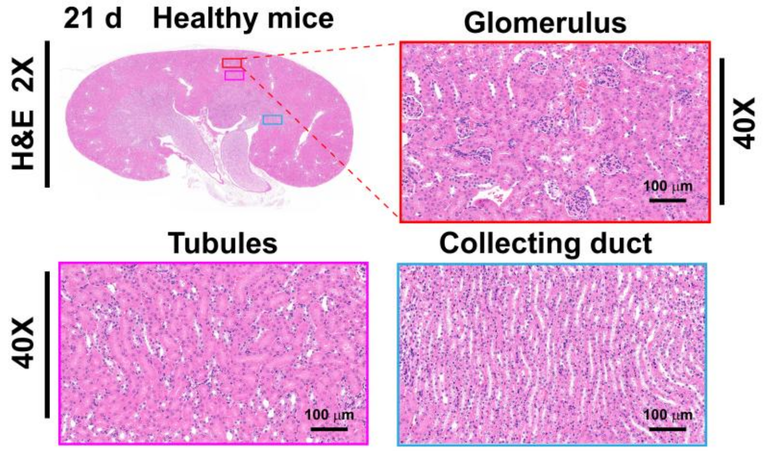

Figure S26. Representative H&E-stained kidney sections from healthy mice at 21d showing preserved histoarchitecture at the whole-organ (2×) and cellular levels (40×; glomerulus, tubules, collecting duct).

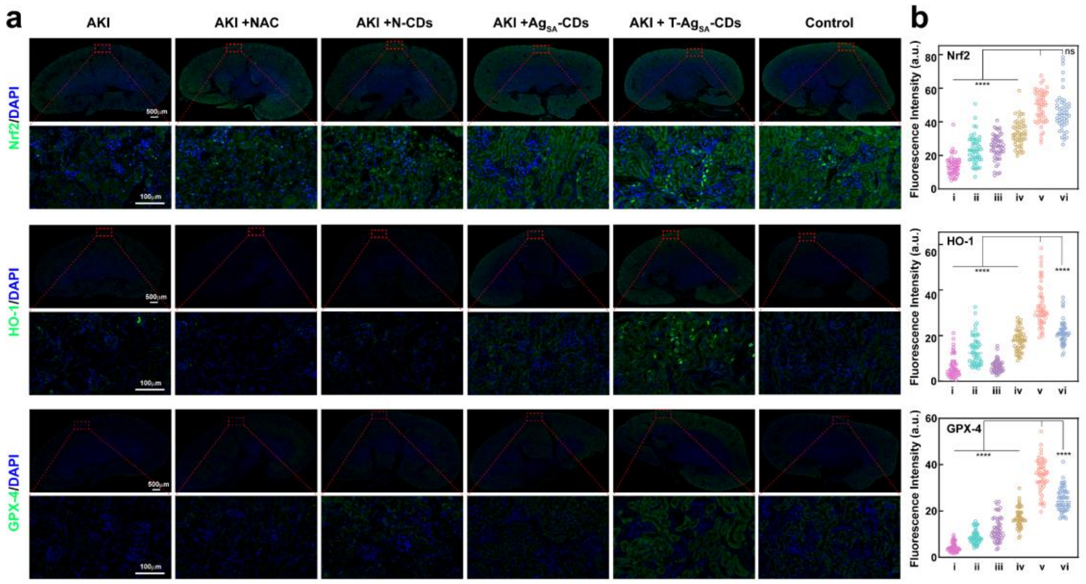

Figure S27. Immunofluorescence analysis of antioxidant signalling activation in kidney tissues. Representative immunofluorescence staining of (top) Nrf2 (green), (middle) HO-1 (green), and (bottom) GPX-4 (green) with nuclear counterstaining by DAPI (blue) in kidney sections from AKI mice treated with PBS (AKI), NAC, N-CDs, Ag_{SA}-CDs, T-Ag_{SA}-CDs, and healthy controls. T-Ag_{SA}-CDs treatment induced robust nuclear localization of Nrf2 and significantly elevated HO-1 and GPX-4 expression compared with all other groups, indicating activation of endogenous antioxidant defense pathways. Scale bars: 500 µm (whole-organ images) and 100 µm (magnified regions). The quantified data are estimated from the individual IF scan using Image J software (Right panel).

Supplementary Tables:

Table S1. Results of the determination of the silver content in different samples via ICP–OES.

Nanozymes	Ag content (wt%)
N-CDs	0.0003
Ag_{SA}-CDs	5.8046

Table S2. Contents of carbon, oxygen, and nitrogen [atom %] in the analyzed samples derived from X-ray photoelectron spectroscopy (XPS)

Nanozymes	C_{1s} (%)	N_{1s} (%)	O_{1s} (%)	Ag_{3d} (%)
N-CDs	70.15	9.99	19.86	-
Ag_{SA}-CDs	60.07	11.26	23.3	5.37

Table S3. EXAFS fitting parameters at the Ag K-edge for various samples (S_0^2=1.0)

Sample	Shell	CN[a]	R(Å)[b]	σ(Å^2)[c]	ΔE_0(eV)[d]	R factor
Ag_{SA}-CDs	Ag-N	10.3±2.1	2.40±0.01	0.0200	7.1	0.0039
	Ag-Ag	10.7±2.1	2.84±0.01	0.0091	5.6	
Ag foil	Ag-Ag	12.0*	2.87±0.01	0.0110	-0.1	0.0127
Ag_2O	Ag-O	1.2±0.3	2.06±0.01	0.0030	9.6	0.0027
Ag_2S	Ag-S	1.9±0.1	2.50±0.01	0.0093	-0.2±0.9	0.0082
	Ag-Ag	3.4±0.5	2.97±0.02	0.0228		
$AgNO_3$	Ag-O	4.7±0.4	2.42±0.04	0.0191	4.7±4.0	0.0179
	Ag-O_1	0.9±0.7	2.93±0.04	0.0087		

(a)CN, coordination number; (b)R, distance between absorber and backscatter atoms; (c)σ^2, Debye-Waller factor to account for both thermal and structural disorders; (d)ΔE(0), inner potential correction; R factor indicates the goodness of the fit. S_0^2 was fixed to 0.95 for Ag. A reasonable range of EXAFS fitting parameters: $0.700 < S_0^2 < 1.000$; CN > 0; $\sigma^2 Å^2 > 0$; $|\Delta E_0| < 10$ eV; R factor < 0.02.

Table S4. Comparison of the typical parameters of SOD-like activities of Ag_{SA}-CDs nanozyme with the reported SOD nanozymes and natural SOD.

**Nanozymes**	**[E] / μg mL^{-1}**	**Assay kit**	**Specific activity /(U mg^{-1})**	**Inhibition rate /%**	**Ref**
Natural SOD	3.03	WST-1 by Dojindo	4743.8	79	[16]
C-dot SOD	2.60		10767	87	
Pt@CNDs	5.21	WST-1 by Dojindo	12,605	81.59	[17]
Re-CNDs	-		496	-	
Fluorescent C-dot SOD	20	WST-1 by Dojindo	4049	-	[18]
C-dots	5	WST-1 by Dojindo	9416	90	[19]
$MnPS_3$	5	WST-1 by Dojindo	721.21	70	[20]
Cu-SAzyme	12.3	WST-1 by Dojindo	448.22	61	[21]
N-CDs	8.0	WST-1 by Dojindo	2763	50	This work
Ag_{SA}-CDs	8.0	WST-1 by Dojindo	11814	98	

[E]: The nanozyme concentration

Table S5. Comparison of the typical parameters of GPx-like activities of Ag_{SA}-CDs nanozyme with the reported GPx nanozymes and natural Gpx.

Nanozymes	[E]	Substrate	K_M (mM)	V_{max} (μMs^{-1})	Ref
**VN_4**	8.58×10^{-7} M	H_2O_2	0.05	7.90	[22]
**Natural GPx**	1.82×10^{-9} M	H_2O_2	0.042	14.1	
**RuO_2NPs**	20 μg mL^{-1}	H_2O_2	0.0172	47.0	[23]
**Ag_{SA}-CDs**	20 μg mL^{-1}	H_2O_2	0.091	12.02	This work

[E]: The nanozyme concentration

References

[1] H. Funke, M. Chukalina, A. C. Scheinost, *J. Synchrotron Radiat.* **2007**, *14*, 426.
[2] B. Ravel, M. Newville, *J. Synchrotron Radiat.* **2005**, *12*, 537.
[3] H. Funke, A. Scheinost, M. Chukalina, *Phys. Rev. B* **2005**, *71*, 094110.
[4] P. Hohenberg, W. Kohn, *Physical Review* **1964**, *136*, B864.
[5] W. Kohn, L. J. Sham, *Physical Review* **1965**, *140*, A1133.
[6] J. VandeVondele, M. Krack, F. Mohamed, M. Parrinello, T. Chassaing, J. Hutter, *Comput. Phys. Commun.* **2005**, *167*, 103.
[7] S. Goedecker, M. Teter, J. Hutter, *Phys. Rev. B* **1996**, *54*, 1703.
[8] C. Hartwigsen, S. Goedecker, J. Hutter, *Phys. Rev. B* **1998**, *58*, 3641.
[9] M. Krack, M. Parrinello, *Phys. Chem. Chem. Phys.* **2000**, *2*, 2105.
[10] J. VandeVondele, J. Hutter, *J. Chem. Phys.* **2007**, *127*, 114105.
[11] J. P. Perdew, K. Burke, M. Ernzerhof, *Phys. Rev. Lett.* **1996**, *77*, 3865.
[12] S. Grimme, J. Antony, S. Ehrlich, H. Krieg, *J Chem Phys* **2010**, *132*, 154104.
[13] J. K. Nørskov, J. Rossmeisl, A. Logadottir, L. Lindqvist, J. R. Kitchin, T. Bligaard, H. Jonsson, *J. Phys. Chem. B* **2004**, *108*.
[14] J. Rossmeisl, Z. W. Qu, H. Zhu, G. J. Kroes, J. K. Nørskov, *J. Electroanal. Chem.* **2007**, *607*, 83.
[15] C. Ling, X. Niu, Q. Li, A. Du, J. Wang, *J Am Chem Soc* **2018**, *140*, 14161.
[16] W. Gao, J. He, L. Chen, X. Meng, Y. Ma, L. Cheng, K. Tu, X. Gao, C. Liu, M. Zhang, K. Fan, D. W. Pang, X. Yan, *Nat Commun* **2023**, *14*, 160.
[17] Y. Zhang, W. Gao, Y. Ma, L. Cheng, L. Zhang, Q. Liu, J. Chen, Y. Zhao, K. Tu, M. Zhang, *Nano Today* **2023**, *49*, 101768.

[18] C. Liu, W. Fan, W. X. Cheng, Y. Gu, Y. Chen, W. Zhou, X. F. Yu, M. Chen, M. Zhu, K. Fan, *Adv. Funct. Mater.* **2023**, *33*, 2213856.
[19] Z. Yan, Y. Zhang, Q. Chen, J. Li, X. Ning, F. Bai, Y. Wang, X. Liu, Y. Liu, M. Zhang, *J. Adv. Res.* **2025**.
[20] C. Zhang, Y. Yu, S. Shi, M. Liang, D. Yang, N. Sui, W. W. Yu, L. Wang, Z. Zhu, *Nano Lett.* **2022**, *22*, 8592.
[21] J. Yang, R. Zhang, H. Zhao, H. Qi, J. Li, J. F. Li, X. Zhou, A. Wang, K. Fan, X. Yan, presented at Exploration **2022**.
[22] S. Zhang, Y. Li, S. Sun, L. Liu, X. Mu, S. Liu, M. Jiao, X. Chen, K. Chen, H. Ma, *Nat. Commun.* **2022**, *13*, 4744.
[23] Z. Liu, L. Xie, K. Qiu, X. Liao, T. W. Rees, Z. Zhao, L. Ji, H. Chao, *ACS Appl. Mater. Interfaces* **2020**, *12*, 31205.
